# Genome-Wide Association (GWAS) Applied to Carcass and Meat Traits of Nellore Cattle

**DOI:** 10.3390/metabo14010006

**Published:** 2023-12-21

**Authors:** Hugo Borges Dos Reis, Minos Esperândio Carvalho, Rafael Espigolan, Mirele Daiana Poleti, Dewison Ricardo Ambrizi, Mariana Piatto Berton, José Bento Sterman Ferraz, Elisângela Chicaroni de Mattos Oliveira, Joanir Pereira Eler

**Affiliations:** 1 Department of Veterinary Medicine, Faculty of Animal Science and Food Engineering (FZEA), University of Sao Paulo, Av. Duque de Caxias Norte, 225, Pirassununga 13635-900, SP, Brazil; minovisk@alumni.usp.br (M.E.C.); mirelep@usp.br (M.D.P.); jbferraz@usp.br (J.B.S.F.);; 2 Department of Animal Science and Biological Sciences, Federal University of Santa Maria (UFSM), Av. Independencia, 3751, Palmeira das Missões 98300-000, RS, Brazil; 3 School of Agricultural and Veterinary Studies (FCAV), São Paulo State University, Jaboticabal 14884-900, SP, Brazil; mapberton@gmail.com

**Keywords:** genomic selection, ssGBLUP, beef cattle, tenderness, intramuscular fat

## Abstract

The meat market has enormous importance for the world economy, and the quality of the product offered to the consumer is fundamental for the success of the sector. In this study, we analyzed a database which contained information on 2470 animals from a commercial farm in the state of São Paulo, Brazil. Of this total, 2181 animals were genotyped, using 777,962 single-nucleotide polymorphisms (SNPs). After quality control analysis, 468,321 SNPs provided information on the number of genotyped animals. Genome-wide association analyses (GWAS) were performed for the characteristics of the rib eye area (REA), subcutaneous fat thickness (SFT), shear force at 7 days’ ageing (SF7), and intramuscular fat (IMF), with the aid of the single-step genomic best linear unbiased prediction (ssGBLUP) method, with the purpose of identifying possible genomic windows (~1 Mb) responsible for explaining at least 0.5% of the genetic variance of the traits under analysis (≥0.5%). These genomic regions were used in a gene search and enrichment analyses using MeSH terms. The distributed heritability coefficients were 0.14, 0.20, 0.18, and 0.21 for REA, SFT, SF7, and IMF, respectively. The GWAS results indicated significant genomic windows for the traits of interest in a total of 17 chromosomes. Enrichment analyses showed the following significant terms (FDR ≤ 0.05) associated with the characteristics under study: for the REA, heat stress disorders and life cycle stages; for SFT, insulin and nonesterified fatty acids; for SF7, apoptosis and heat shock proteins (HSP27); and for IMF, metalloproteinase 2. In addition, KEGG (Kyoto encyclopedia of genes and genomes) enrichment analysis allowed us to highlight important metabolic pathways related to the studied phenotypes, such as the growth hormone synthesis, insulin-signaling, fatty acid metabolism, and ABC transporter pathways. The results obtained provide a better understanding of the molecular processes involved in the expression of the studied characteristics and may contribute to the design of selection strategies and future studies aimed at improving the productivity of Nellore cattle.

## 1. Introduction

The beef industry plays a vital role in the global economy, and Brazil stands out as one of the largest beef exporters worldwide. The proportion of beef in Brazilian exports has averaged 6.5% of the total value exported in recent years [1]. While the quantity of beef exported is noteworthy, it is important to address the increasing consumer demands. Consumer expectations and preferences for high-quality beef are on the rise. Therefore, there is room for improvement in the quality characteristics of beef, particularly in Zebu cattle, which are the main source of exported beef. Notably, key quality attributes such as tenderness and marbling directly impact meat quality.

Developing desirable meat quality attributes is crucial to increasing meat consumption. The tenderness of meat, for example, is considered a crucial characteristic for the consumer, as it is the most relevant sensory aspect when choosing the product [2]. Another characteristic, intramuscular fat (IMF), is responsible for promoting juiciness and tenderness, making the meat more pleasant to consume. Additionally, IMF contributes to the flavor and aroma of grilled meat through the combination of nonvolatile compounds, volatile acids, aromatic acids, and fatty acids released during cooking [3,4].

According to [5], the rib eye area (REA) is a body composition indicator capable of determining an animal’s meat content. Moreover, the REA can influence carcass classification/evaluation and, consequently the final meat price. Additionally, subcutaneous fat thickness (SFT) is another important trait that affects meat yield, flavor, and juiciness [6]. SFT also provides protection against mechanical and thermal shocks during the production chain, indirectly influencing meat quality. Collectively, the REA, SFT, and IMF represent the most important measurable meat characteristics that can easily be tracked and evaluated, and with potential applications in meat improvement.

To enhance meat quality, it is essential that bovine genetic improvement programs incorporate the aforementioned attributes. Understanding the genetic basis of carcass and meat quality in Nellore cattle is crucial for improving breed productivity. The use of single-nucleotide polymorphism (SNP) chips, for example, enables us to explore molecular markers on DNA associated with meat quality by genome-wide association studies (GWAS). This approach can correlate SNP allele frequencies with phenotypic traits, including genes related to muscle growth, development, and meat tenderness [7].

To further enhance the accuracy of these analyses, the single-step genomic best linear unbiased prediction (ssGBLUP) method is often employed. This method combines information on pedigree, phenotype, and genotype data, which leads to greater identification and precision in estimates using a relationship matrix [8]. Our hypothesis is that GWAS by ssGBLUP can shed light on the meat characteristics evaluated in our study. Thus, the aim is to identify genomic regions and candidate genes associated with IMF, the REA, SF7, and SFT. For these reasons, we selected an SNP database of 2431 animals with known REA (2417), SF7 (2368), SFT (2431), and IMF (1854) information. These data were selected and used to perform the GWAS technique, and subsequently to analyze the enrichment of pathways to improve the accuracy of biological processes behind meat quality.

## 2. Materials and Methods

### 2.1. Animals, Slaughter and Genotyping

The animals used in this study were male (N = 2458) and female (N = 12) Nellore cattle from farms in the western region of the state of São Paulo belonging to the company Agropecuária CFM Ltda (São José do Rio Preto, Brazi). All animals were raised under similar management conditions (under pasture conditions) until 18 to 22 months of age, and then fed in feedlots until slaughter at between 22 and 26 months of age, receiving a diet composed of grains. Data were recorded between the years 2006 and 2013. The animals were slaughtered at a mean age of 24 (±4) months in a commercial slaughterhouse belonging to the Marfrig group. The average hot carcass weight observed was 270.6 kg ± 28.7. Slaughter was carried out in accordance with humane slaughter procedures.

During deboning, 24 h after slaughter, the rib eye area (REA) and subcutaneous fat thickness (SFT) were measured in the *Longissimus thoracis* muscle between the 12th and 13th rib of the left half carcass. The REA was dimensioned with a transparent grid of points superimposed on a muscle cross-section (cm^2^), and SFT was measured with the aid of a ruler graduated in millimeters [9]. Intramuscular fat (IMF) analysis was performed on approximately 100 g of freeze-dried and ground beef, following the method of [10]. For the analysis of tenderness by shear force, an approximately 2.5 cm thick steak collected in the *Longissimus thoracis* muscle between the 12th and 13th ribs was kept in a cold chamber for 7 days at a temperature between 0 °C and 2 °C after being vacuum packed. The determination of tenderness was performed using a Warner–Bratzler blade, as recommended by the American Meat Science Association [11]. Shear force, or tenderness (SF7), was determined by averaging the cylinder values in Newton (N). Among all slaughter animals, a total of 2470 animals were included in the SNP database, with 662,122 individual SNPs available in addition to pedigree information.

The database used contained information on 2470 male and female animals. Of these, a total of 2181 animals were genotyped using the Illumina BovineHD BeadChip (Illumina Inc., San Diego, CA, USA), totaling 777,962 SNPs. For each sample, the overall quality of genotyping was assessed through the genotype determination rate (call rate), defined as the ratio between calls and the total number of markers. Samples with a call rate lower than 0.9 (<90%) of the determined genotype were discarded. Furthermore, for GWAS, quality control of SNP markers consisted of excluding those with unknown genomic positions, those located on the sex chromosomes, monomorphic SNPs, markers with a deviation of the Hardy–Weinberg equilibrium greater than 0.17, and SNPs with a minor allele frequency (MAF) less than 0.05. Only SNPs on autosomal chromosomes and with a known position according to the ARS UCD 1.2 bovine genome were taken into consideration. This process was carried out with the help of the PREGSF90 program, using scripts developed for this purpose [12], which resulted in a data set containing 468,321 SNPs and 2181 animals.

### 2.2. Quantitative Genetic Analysis

The fixed effects included in the model for both traits studied were contemporaneous group and animal age at slaughter as a covariate. As a random effect, the animals’ genetic effects were included, in addition to the residual effects. Contemporaneous groups (CGs) of animals included individuals born on the same farm and in the same year, exhibiting the same sexual condition, and included in the same group for slaughter. CGs that contained fewer than three observations and those that contained observations that deviated ±3 standard deviations from the mean of the group were eliminated. Genetic parameters, in addition to variance components, were estimated using REMLF90 version 1.86 software, which is from the BLUPF90 family, and ssGWAS computer programs were also used [12,13]. The statistical model used is represented in the following matrix:

(1) $$y=X\beta +Z_{a}+\mathrm{e}$$

where *y* represents the vector of observations, *β* is the vector of fixed effects, *a* is the vector of direct additive genetic effects, *X* is the known incidence matrix, *Z* represents the incidence matrix of random direct additive genetic effects (associates vector *β* with vector *y*), and e represents the residual effect.

### 2.3. Genomic Association Analysis

For the implementation and execution of GWAS, we used the single-step GBLUP method (ssGBLUP). This method consists of modifying BLUP by replacing the numerator A1 with H1 in the matrix [14]:

(2) $$H^{-1}=A^{-1}+\left[0\;0\;0\;G^{-1}-A_{22}^{-1}\;\right]$$

The element *A*^−1^ is the inverse of the additive relationship matrix, $A_{22}^{-1}$ refers to the inverse of the additive relationship matrix and includes only the genotyped animals, while *G*^−1^ is the inverse of the genomic relationship matrix. The genomic matrix can be formulated as follows [15]:

(3) $$G=ZDZ^{\prime }q$$

In this case, “*Z*” represents a gene array adjusted for allelic frequency; “*D*” is a matrix referring to the weight of the SNP (initially *D* = *I*); “*q*” is the weighting factor. According to [16], this factor ensures that the mean diagonal “*G*” is close to that of A22. Effects and SNP weights for GWAS were derived as follows, as presented in [17]:

1. Let *D = I* in the first step;
2. Calculate *G = ZDZ′q*;
3. Calculate GEBVs for the entire data set using ssGBLUP;
4. Convert GEBVs to SNP effects.

(4) $$\hat{u}=\frac{\sigma_{u}^{2}}{\sigma_{a}^{2}}DZ^{\prime }G^{*-1}{\hat{a}}_{g}=DZ^{\prime }{\left[ZDZ^{\prime }\right]}^{-1}{\hat{a}}_{g}$$

where *â_g_* is considered to be the GEBV of the animals that were also genotyped.

5. Calculate the weight for each SNP as follows: $d_{i\;}={û}_{i}^{2}2pi(1-p_{i})$*,* where *i* represents the *i*-th SNP;
6. Normalize SNP weight to preserve constant variance in total genetics.
7. Loop to step 2.

The density of SNPs was calculated iteratively in a loop. Iterations of step 4 were able to raise the weights of SNPs with large effects and minimize those with small effects. The percentage of genetic variance explained by the *i*-th region was calculated according to the equation below:

(5) $$\frac{Var\left(a_{1}\right)}{\sigma_{u}^{2}}=x100=\frac{Var\left(\sum_{j=1}^{1\mathrm{Mb}}Z_{jûj}\right)}{\sigma_{u}^{2}}$$

*a_i_* represents the genetic value of the *i*-th region consisting of 1 Mb continuous adjacent SNPs, $\sigma_{a}^{2}$ represents total genetic variance, *Z_j_* is the gene content vector of the *j*th SNP for all animals, and *ûj* represents the effect of the *i*-th SNP marker located in the *i*-th region.

### 2.4. Enrichment Analysis

To determine the possible regions comprising QTLs as a criterion for the investigation of genes, genomic regions that explained values equal to or greater than 0.5% of additive genetic variance were chosen. For a broad genetic investigation, these regions were expanded by 500 Kb at each end and the genes present there were identified using the R software package v.4.1.2. [18] called Genomic Annotation in Livestock for positional candidate Loci (GALLO) [19]. The same package was used for functional enrichment based on Medical Subject Headings (MeSH) terms. Only terms with a false discovery rate (FDR)-adjusted *p* value < 0.05 were considered significant in this study.

The same gene list was explored using the KEGG database through Clusterprofiler package [20]. For this step, we performed the analysis using ENTREZID code with groups as conditions. We used the functions enrich KEGG, enrichGO, and compareCluster to explore differences in conditions and compared functional enrichment for gene ontology. We considered a *p*-value < 0.05 for enrichment. Graphics were constructed using enrichplot package [21]. Additional packages were used to tidy data, such as tidyr [22], dplyr [23], and readr [24]. This resource provided information on biological processes (BPs), cellular components (CCs), and molecular functions (MFs).

## 3. Results

### 3.1. Description of Phenotypic Data and Heritability

The descriptive statistics and heritabilities of the analyzed phenotypic data are shown in Table 1. The heritabilities (*h*^2^) ± *SD* estimated without genomic information ranged from moderate to low (0.11 ± 0.05 for REA; 0.20 ± 0.07 for SFT; 0.18 ± 0.08 for SF7; and 0.21 ± 0.09 for IMF). The heritabilities (*h*^2^*g*) ± *SD* estimated using genomic information also showed variation between moderate and low values (0.14 ± 0.03 for REA; 0.12 ± 0.03 for SFT; 0.10 ± 0.04 for SF7; and 0.21 ± 0.05 for IMF).

### 3.2. Genome-Wide Association Study and Identification of Candidate Genes

The proportion of additive genetic variance explained by nonoverlapping genomic windows of 1 Mb of SNPs was estimated for all analyzed traits, considering 29 autosomal chromosomes. Only the genomic windows that explained at least 0.5% of the additive genetic variance for each trait were considered; thus, an average of 255 SNPs were identified per window. The results obtained are described in Table 2. 

For REA, 3715 genomic windows were estimated, of which 1891 presented percentage values of genetic variance different from zero, and only 10 explained more than 0.5% of additive genetic variance for the trait. For the SFT phenotype, 3577 windows were estimated, of which 1891 genomic windows had a percentage of additive genetic variance greater than zero; of these, 11 explained more than 0.5% of genetic variance for the trait. For SF7, 2981 genomic windows were estimated, and of these, 1891 had a percentage of additive genetic variance different from zero, of which six explained more than 0.5% of additive genetic variance for SF7. Finally, 3061 windows were estimated for IMF, and of this estimated total, 1872 genomic regions had a percentage of genetic variance above zero, of which only seven explained more than 0.5% of genetic variance for the trait of IMF.

**Figure 1:**
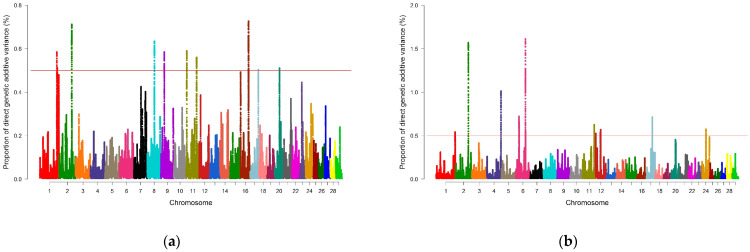
Manhattan plots of the posterior means of the percentages of genetic variance explained by each window of 1 Mb SNPs along the 29 autosomal chromosomes for the analyzed carcass quality traits: (**a**) rib eye area (REA); (**b**) subcutaneous fat thickness (SFT). Red lines indicate the threshold for considering significant SNP windows (0.5%). Colors indicate different chromosomes.

**Figure 2:**
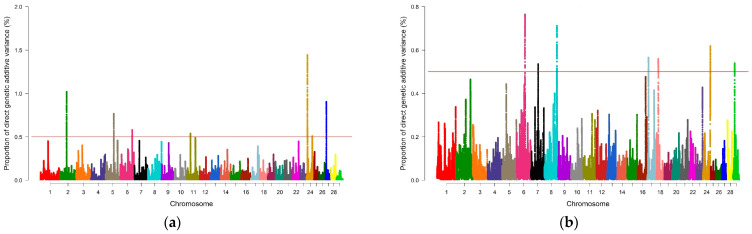
Manhattan plots of the posterior means of the percentages of genetic variance explained by each window of 1 Mb SNPs along the 29 autosomal chromosomes for the analyzed carcass quality traits: (**a**) shear force at 7 days’ ageing (SF7); (**b**) intramuscular fat (IMF). Red lines indicate the threshold for considering significant SNP windows (0.5%). Colors indicate different chromosomes.

The Manhattan plots shown in Figure 1 and Figure 2 report the proportion of genetic variance explained by the windows of SNPs distributed over the 29 chromosomes for the characteristics of REA, SFT, SF7 and IMF, respectively. The points located above the red line represent the regions capable of explaining at least 0.5% of additive genetic variance. The results indicated significant regions on chromosomes 1, 2, 4, 5, 6, 7, 8, 9, 11, 12, 16, 17,18, 20, 24, 27, and 29 for the characteristics of REA, SFT, SF7, and IMF. These genomic regions were used to search for candidate genes related to each trait.

These regions were used to search for candidate genes related to each trait, which are shown in Table 3, Table 4, Table 5 and Table 6 for REA, SFT, SF7, and IMF, respectively.

### 3.3. Functional Analysis and Pathway Enrichment

**Figure 3:**
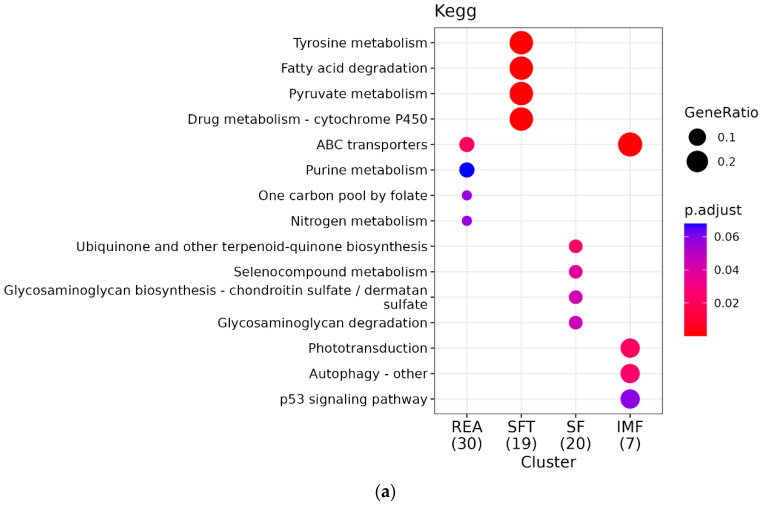

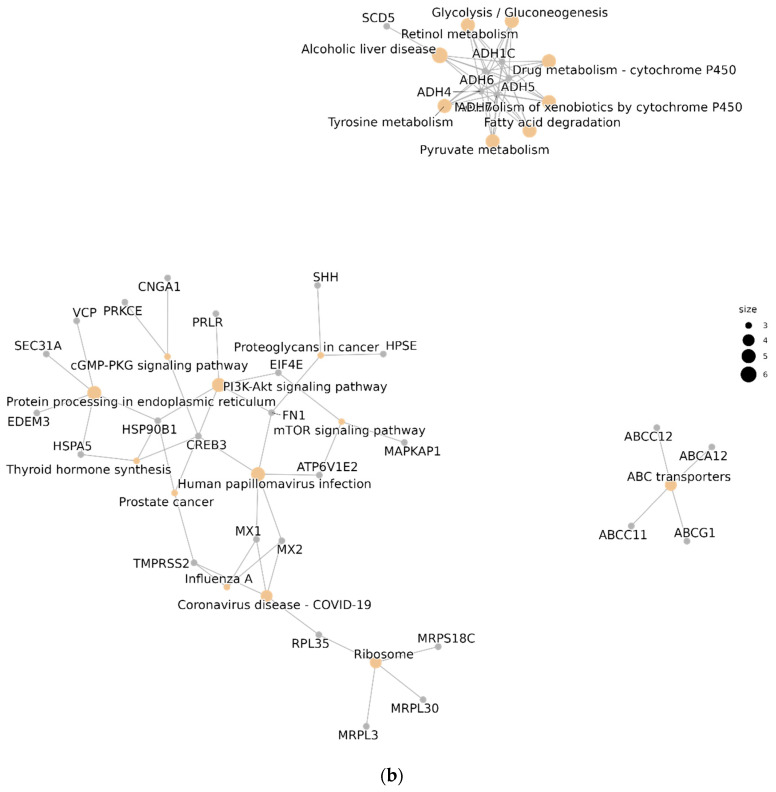
KEGG enrichment analysis: (**a**) comparative pathway analysis for REA, SFT, SF, and IMF groups. “GeneRatio” indicates the proportion of genes within a specific pathway relative to the total number of genes enriched (expressed as a fraction in parentheses beside the group name). The “P.adjust” value represents statistical significance, with smaller values depicted in red and larger values in blue. (**b**) KEGG network analysis of enriched genes. Nodes in the network represent pathways, interconnected with genes belonging to those pathways. Central pathways or genes with higher connectivity are highlighted as they play a more pivotal role in this network. These improvements make the text clearer and more concise, ensuring that the information is presented in a more organized and reader-friendly manner. Size represents the number of genes in that pathway.

Network enrichment was performed for all phenotypes and indicated a set of ADH family genes modulating a large number of important pathways such as glycolysis, fatty acid degradation, pyruvate metabolism, and tyrosine metabolism. Another highlight is the ribosome pathway, which appears to be modulated by three genes from the same group called mitochondrial ribosomal proteins (MRP), which are ribosomal proteins important for the proper biogenesis of ribosomes [25]. In addition, genes such as *HSP90B1* and *HSPA5* are centrally located in the graph, indicating their participation in several pathways simultaneously (Figure 3), which can be explained by their function as molecular chaperones responsible for protein folding, assembly, translocation, and degradation in the stabilization of proteins and membranes [26].

Functional enrichment analysis for MeSH terms revealed 47 terms related to REA, 88 terms related to SFT characteristics, 45 related to SF7, and 34 terms related to IMF (Supplementary Archive S1). Among the highly significant terms found for REA, the following stand out: heat stress disorders, gestational age, amino acid substitution, adenosine monophosphate, and fibronectin receptors. As for the SFT characteristic, terms such as nonesterified fatty acids, cholesterol ester transfer proteins, cholesterol esters, VLDL lipoproteins, body patterning, lipoproteins, and epithelial cells stand out for their direct or indirect associations with the studied phenotype. For SF, a predictor of meat tenderness, some highly associated terms are worth mentioning, such as HSP27 heat shock proteins, purinergic P2Y2 receptors, heme oxygenase-1, lipoprotein lipase, and stress fibers. In relation to IMF, the terms propionates and sodium deserve to be highlighted.

**Figure 4:**
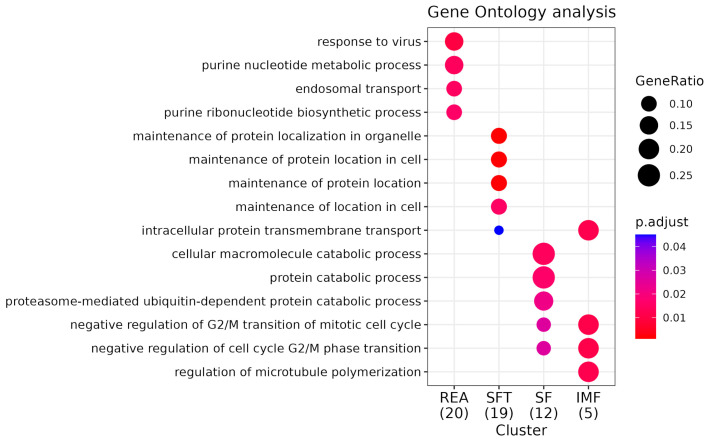
GO enrichment analysis for biological processes (BPs). Comparative pathway analysis of REA, SFT, SF, and IMF groups. “GeneRatio” indicates the proportion of genes within a specific pathway relative to the total number of genes enriched (expressed as a fraction in parentheses beside the group name). The “P.adjust” value represents statistical significance, with smaller values depicted in red and larger values in blue. BP is indicative of the processes behind the gene function.

**Figure 5:**
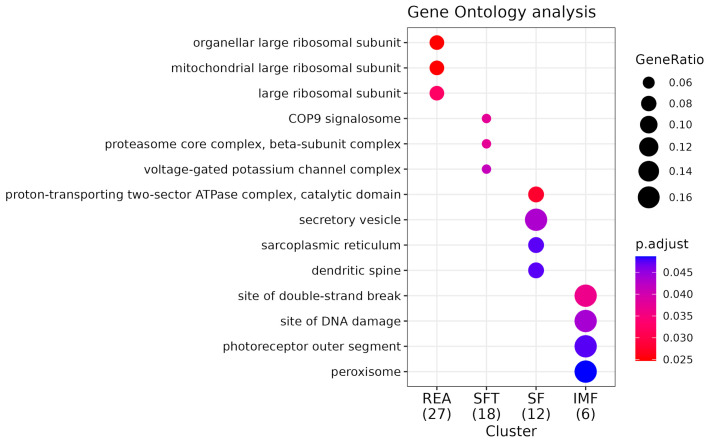
GO enrichment analysis for cellular components (CCs). Comparative pathway analysis of REA, SFT, SF, and IMF groups. “GeneRatio” indicates the proportion of genes within a specific pathway relative to the total number of genes enriched (expressed as a fraction in parentheses beside the group name). The “P.adjust” value represents statistical significance, with smaller values depicted in red and larger values in blue. CCs suggest where a function occurs, and molecular function indicates the action of a product.

**Figure 6:**
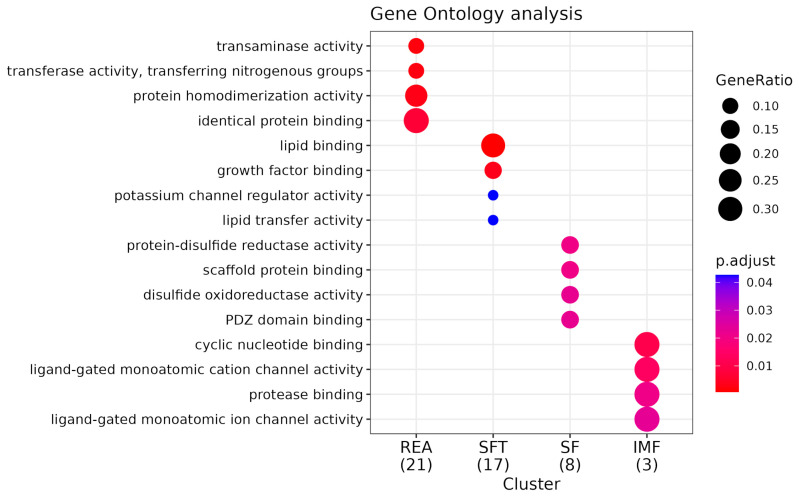
GO enrichment analysis for molecular function (MF). Comparative pathway analysis of REA, SFT, SF, and IMF Groups. “GeneRatio” indicates the proportion of genes within a specific pathway relative to the total number of genes enriched (expressed as a fraction in parentheses beside the group name). The “P.adjust” value represents statistical significance, with smaller values depicted in red and larger values in blue. Molecular functions are the actions of a product.

Kegg enrichment identified numerous statistically significant pathways associated with the characteristics addressed in this work. However, some of them stand out for their association with the phenotypes addressed in this work (Supplementary Archive S1). For REA, these are growth hormone synthesis, secretion, and action (bta04935) and ribosome pathway (bta03010). The enriched gene for bta04935 was *CREB3* and for the ribosome pathway, the genes were *MRPL30* and *MRPL3*. During enrichment analysis using Gene Ontology, we identified 35 biological process (BPs) (Figure 4), among which the following stand out: endoplasmic reticulum stress response (GO:0034976) and ribosomal biogenesis (GO:0042254). Additionally, we found three significant cellular components (CCs) (Figure 5) highlighting the large ribosomal subunit (GO:0015934), due to its relationship with the phenotype in focus. We further identified ten molecular function (MF) pathways (Figure 6); however, the most important was GTPase regulator activity (GO:0030695) (Supplementary Archive S2). 

For SFT, we observed distinct pathways, such as the fat digestion and absorption (bta04975, *MTTP*) and insulin-signaling pathways (bta04910, *EIF4E*) For SFT, we identified 39 significant table BPs, with an emphasis on negative regulation of the lipid biosynthetic process (GO:0051055), organization of subunits of the protein–lipid complex (GO:0071825), and intermembrane transfer of lipids (GO:0120009). Three distinct CCs were identified but none in direct association with SFT. Finally, among the five identified MFs, we observed functions associated with lipid binding (GO:0008289) and lipid transfer activity (GO:0120013), probably due to their relationship with the associated characteristics (Supplementary Archive S3).

In the case of SF, the pathways with the best relationship were biosynthesis of unsaturated fatty acids metabolism (bta01040) and fatty acid metabolism (bta01212). For both pathways, the enriched gene was *SCD5*. We found 37 BP pathways (Supplementary Archive S3), and among these, some stand out for their association with the characteristic studied, such as mesoderm development (GO:0007498). We observed six CC pathways, with an emphasis on the sarcoplasmic reticulum (GO:0016529) due to its high relationship with the evaluated tenderness phenotype. Finally, we noticed that scaffold protein binding (GO:0097110) stands out among the 14 MF pathways in association with SF.

For IMF, we found a total of 12 enriched pathways (Supplementary Archive S3), but the most important pathway was ABC transport (bta02010), with the genes *ABCC11* and *ABCC12*. Furthermore, we observed 38 BP pathways (Supplementary Archive S3). The most relevant were lipid oxidation (GO:0034440) and lipid modification (GO:0030258). Additionally, we noticed that five CC pathways were present; however, we highlight the peroxisome (GO:0005777) due to its high importance for lipid metabolism. Finally, with regard to MF, 11 functions associated with IMF stand out in our study; however, we chose to emphasize the function of gated channel activity (GO:0022836) due to its relevance to the phenotype in question.

## 4. Discussion

### 4.1. Description of Phenotypic Data and Heritability

Mean heritability estimates for the studied phenotypes obtained by both methods did not improve when genomic information was included. This may have occurred because the number of animals with available phenotypes was slightly higher than the number of genotyped animals, as was also reported in another published study [27]. However, ref. [28] suggests that if available, genomic information should be included in variance component analysis, especially in genetic covariance value, when a trait has a limited number of observations and a high percentage of unknown parents. Additionally, genomic information can improve the accuracy of the studied genetic values when compared to those obtained only based on phenotype and/or pedigree.

In a study conducted by [29] using Nellore animals and the ssGBLUP method, the authors demonstrated a heritability of (0.34 ± 0.02) for REA, which was higher than that found in our study (Table 1). Similarly, ref. [30] conducted a genetic analysis of carcass and meat traits in Nellore animals, reporting a heritability value of 0.21 for SFT. This finding was similar to that found in our study without the inclusion of genomic data, but higher than the value obtained with the use of genomic data (0.12). For intramuscular fat, a study carried out by [31] obtained a heritability value of (0.29 ± 0.16). This value was higher than the one observed in our study (0.21 ± 0.05). These differences in heritability can be explained by numerous factors: (1) difference in the population lineages of Nellore animals present in Brazil; (2) the consistency of the pedigree; (3) number of animals used in the studies.

### 4.2. Genome-Wide Association Study and Candidate Genes

REA measurements have a high correlation with muscle growth and deposition. Therefore, they have great importance for the meat industry. In our study, we identified 80 genes within the genomic windows associated with REA, with some genes standing out in GO based on their biological processes (PRDM15, TPM2, and TESK1). The *PRDM15* gene, which encodes the PR/SET domain 15 protein, directly regulates the *Mitogen-activated protein kinase* (MAPK)-signaling pathway. This pathway is involved in muscle growth and myogenesis, which may be influenced by stress and physical activity [32]. Ref. [33] found an association between the *PRDM15* gene and the characteristic of loin development in Charolais cattle, as reported in this study.

Still, *TPM2* (Tropomyosin 2) and *TESK1* (*Testis Associated Actin Remodeling Kinase 1*), found on chromosome 8, have functions associated with cytoskeletal organization and may be of great interest for REA. Ref. [34] reported the presence of the *TPM2* gene in association with skeletal muscle structure in cattle.

The SFT and REA characteristics are important for carcass quality. In our study, we observed a total of 51 candidate genes associated with SFT, some of which are noteworthy due to their potential influence on the phenotype. One of them is the *MTTP* gene (*Microsomal triglyceride transfer protein large subunit*), which is responsible for encoding the microsomal triglyceride transfer protein and is directly linked to lipid metabolism. This may explain subcutaneous fat deposition variations in bovine animals [35]. A study conducted by [36] on swine also found an association between this gene and the SFT phenotype, concluding that *MTTP* directly affects the metabolism of fatty acids and that with it, subcutaneous or intramuscular fat thickness becomes totally compromised. Another candidate gene observed in association with SFT is *PAXIP1* (*PAX Interacting Protein 1*), located on bovine chromosome number 4. This gene is part of the PAX gene family, which, among other functions, plays an important role in maintaining the stability of the genome, and is associated with the progression of adipose tissue over time, from its formation to its mature structure. According to [37], *PAXIP1* forms a complex with *MLL3* (*mixed-lineage leukaemia 3*) and the related protein *MLL4*, increasing H3K4me3 in the adipogenic promoters *PPARG* and *CEBPA*. In addition, a deletion in *PAXIP1* inhibits preadipocyte differentiation in white and brown fat.

For the SF7 phenotype, a quantitative trait of extreme importance for the acceptability of meat products on the consumer market, three genomic regions with significant percentages of genetic variance were located, in which we found candidate genes with possible relevant functions associated with SF. One of these genes is *HSP90B1* (Heat shock protein 90 kDa beta member 1), responsible for encoding a heat shock protein that has relevance because apoptosis is related to the tenderness of meat; more specifically, it is related to the enzymes acting during apoptosis, called caspases, which are responsible for degrading calpastatin. Calpastatin has an antagonistic action to calpain, due to which, as the two are enzymes responsible for the degradation of postmortem muscle fibers, it is understood that the presence of the *HSP90B1* gene can influence the tenderness of meat [38,39]. A study conducted by [40] found an association between the *SCD5* gene and the tenderness of meat in a population of cross-bred cattle (Angus–Brahman). In our work, we identified the same gene, located on chromosome 6, as a candidate for association with SF. The *SCD5* gene is responsible for encoding the protein *Stearoyl-CoA desaturase 5*, which is related to the formation of monounsaturated fatty acids from saturated fatty acids. The presence of fatty acids in meat can make it more tender.

In terms of the IMF phenotype, we found four distinct genomic regions with candidate genes on chromosomes 6, 8, 18, and 29. Among the candidate genes observed, *LONP2* stands out, responsible for encoding the peroxisomal *Lon peptidade 2 protein*, which is directly associated with the organization of peroxisomes. Peroxisomes are organelles located in the cytoplasm that perform numerous functions, including lipid metabolism, breaking down fatty acid chains (β-oxidation). Ref. [41] recorded an association between the *LONP2* gene and fatty acid metabolism in taurine animals. These facts corroborate the conclusion of a possible role of this gene in the IMF characteristic.

### 4.3. Functional Analysis and Pathway Enrichment

#### 4.3.1. Functional Analysis

The term heat stress disorders is related to the effects of heat stress and has a crucial role in animal metabolism, being associated with their thermoregulation [42]. Animals suffering from heat stress have decreased feed intake, growth, and production efficiency [43]. Mutations in the prolactin receptor (*PRLR*) gene have been reported to affect the temperature tolerance of cattle and may reduce the impact of heat stress on production [44]. The maintenance of skeletal muscle tissue homeostasis through protein turnover is essential; therefore, the identification of pathways related to the immune system and ubiquitins among the MeSH terms found is justified. Ref. [45] also identified pathways related to muscle differentiation and ubiquitination of proteins associated with REA, corroborating our results.

In terms of the SFT phenotype, insulin was observed in our study. It is linked to the regulation of glucose metabolism, directly suppressing endogenous glucose production and indirectly suppressing lipolysis. Ref. [46] reported that plasma insulin concentration is positively correlated with carcass adiposity. Another term observed was nonesterified fatty acids, nonesterified or “free” fatty acids, which are extremely important for lipid metabolism. A study conducted by [47] on swine suggests that nonesterified fatty acids can be characterized as biomarkers for meat quality characteristics, such as intramuscular fat content and fatty acid composition in animals in the finishing phase.

For SF, the main term we found to be associated with this characteristic was HSP27 heat shock proteins. So-called heat shock proteins are important for animal physiology, as they have a protective role, increasing cell survival during periods of stress. According to [48], the HSP27 protein seems to delay the onset of proteolysis; however, as the maturation process progresses to 7 and 14 days after slaughter, the presence of such proteins may contribute to the tenderness of the meat.

Regarding the IMF phenotype, the term matrix metalloproteinase 2 was identified, referring to a family of endopeptidases that are involved in the remodeling of extracellular matrix components [49]. Extracellular matrix enzymes are excellent candidates for remodeling body fat [50]. According to [51], matrix metalloproteinases cleave extracellular matrix components such as collagen and laminin and free up space between cells, allowing for cell mobility. IMF is located in intramuscular connective tissue, so the intramuscular remodeling of this tissue by matrix metalloproteinases is extremely important. In addition, the synthesis of new extracellular matrix components by fibroblasts is critical for the development of fat storage between and within muscle fascicles [52].

#### 4.3.2. Network Enrichment

Network enrichment showed us a cluster of genes from the alcohol dehydrogenase (*ADH*) family related to numerous pathways. The genes detected in our work, for example alcohol dehydrogenase 6 (*ADH6*), alcohol dehydrogenase 5 (*ADH5*), and alcohol dehydrogenase 4 (*ADH4*), were mentioned in several works involving beef cattle. A work carried out by [53] highlighted both *ADH4* and *ADH6* enriched in KEGG pathways for retinol metabolism in Angus animals. A study conducted by the author highlighted that the *ADH6* gene was one of the central genes when studying [54] the characterization of key molecular signatures related to feed efficiency for meat quality in different bovine breeds. Furthermore, ref. [55] reported that the *ADH5* gene may be related to selection for growth in Nellore cattle. All these *ADH* genes were detected in our work, but enriched for different pathways, such as FA metabolism, pyruvate metabolism, and glycolysis. The sum of this information leads us to believe that this group of genes is highly important for the metabolism of cattle in general, and that understanding their expression can generate a positive impact on the production chain of beef cattle.

The enrichment of KEGG networks showed us that the ribosome pathway was enriched with some genes from the mitochondrial ribosomal protein (*MRP*) family. One of them was the *MRPL3* gene, also enriched in a work proposed by [56], involving animals of the Sahiwal breed (*bos indicus*), where it was associated with milk composition in these animals. Another gene from this family identified in the ribosome pathway in our study was *MRPL30*. This gene has high importance for ribosomes and was also identified in a work proposed by [57], where the author reports that this gene is involved in translation and in the structural constituents of ribosomes in *bos taurus* animals. In addition, genes from the heat shock protein (HSP) family were identified more centrally in our network enrichment, evidencing their participation in the enriched pathways. *HSPs* play an essential role in cell protection and heat tolerance during heat stress, providing protein folding and refolding, promoting protein aggregation, and overcoming potentially harmful interactions [58]. A study conducted by [59], evaluating the vitrification method for in vitro and in vivo survival of *bos taurus* embryos, demonstrated that this technique can promote the transcription of the *HSPA5* gene in response to cellular stress. Although the focus of our work is not the same, we also found the *HSPA5* gene, demonstrating that, in some way, the stress response can affect the metabolism of enriched pathways associated with this gene in our study.

#### 4.3.3. Pathway Enrichment KEGG

Understanding the factors that affect animal growth and development can provide tools to optimize profitability and meat quality [60,61]. The yield of meat cuts is mainly due to the deposition of muscle and adipose tissue in an animal’s body [62]. The characteristics addressed in this work, such as REA and SFT, are productive indicators and are widely used to predict the amount of meat in the carcass, and the SF7 and IMF characteristics are highly correlated with meat quality [63]. Numerous metabolic processes are involved in the increase in the number of muscle fibers, called hyperplasia, and in the process of muscle hypertrophy, such as the coordinated expression of myogenic regulatory factors and genes in addition to metabolic pathways, from embryonic development to the adult life of the animal [64]. These elements are responsible for the deposition of muscle and adipose tissue, which, in addition to promoting greater meat volume, can modify the composition of the tissue, altering the sensory qualities of the meat, such as flavor and tenderness.

The enrichment analysis carried out identified relevant pathways (KEGG) related to each of the four characteristics addressed. Starting with REA, the growth hormone synthesis, secretion, and action pathways must be mentioned because growth hormone (GH) plays a key role in cell growth, development, and metabolism throughout the body [65]. Once released into the circulation, GH binds to its receptor (GHR) on the cell surface in target tissues such as liver, bone, muscle, and adipose tissue. According to [66], genotyping animals by the GH gene has scientific relevance as it allows us to establish the genetic potential of meat productivity in animals with indicators such as live weight gain, carcass weight, meat yield, and marbling. GH-releasing hormone (GHRH) interacts with a G protein-coupled receptor on somatotroph cells to activate the cyclic adenosine monophosphate (cAMP)-signaling pathway, leading to increased transcription and release of GH mRNA.

The CREB3 gene enriched for this pathway encodes a transcription factor that is a member of the leucine zipper family of DNA-binding proteins. This protein binds to the cAMP response element and regulates cell proliferation. A work carried out by [67] in mice demonstrated that CREB3 family genes are crucial for GHRH expression and that failure to increase GH is mediated by small levels of active nuclear N-terminal CREB3 protein fragments, showing that the gene can act in regulating growth.

Another pathway of great importance found was the ribosome pathway. This pathway is essential for the coordination of protein synthesis, growth, and cell proliferation. Skeletal muscle tissue is primarily determined by elevations in protein synthesis, which in turn is regulated by ribosomal content in muscle [68]. Therefore, this pathway may be related to the deposition of skeletal muscle tissue, which can be verified by measuring the rib eye area. Enrichment analysis verified biological processes relevant to REA, the first being the endoplasmic reticulum stress response (GO:0034976). The endoplasmic reticulum (ER) has several functions, including translocation of proteins (such as secretory proteins) across its membrane, integration of proteins into the membrane, folding and modification of proteins in the ER lumen, synthesis of phospholipids and steroids in the cytosolic region of the ER membrane, in addition to the storage of calcium ions from its lumen and their regulated release in the cytosol [69]. In a study in mice (*C2C12*), ref. [70] proposed that ER stress-induced apoptosis is capable of improving the quality of myoblasts, concluding that this fact may be favorable for myogenesis.

The second biological process identified for REA was ribosomal biogenesis (GO:0042254). As previously mentioned, the ribosome is a supramolecular structure composed of ribonucleoproteins that plays a central role in the translation mechanism, converting mRNA into proteins. Ribosome formation is a crucial factor for cells’ protein synthesis capacity, thus having a key role in controlling cell growth in eukaryotic organisms [71]. A work conducted by [72] proved the importance of ribosomal biogenesis in the control of skeletal muscle mass in response to anabolic stimuli, that is, through nutrition or drugs and under catabolic conditions. Another relevant piece of information for the REA characteristic was one of the genes enriched for this BP, namely the biogenesis of *BRX1* ribosomes (*BRIX1*). In a study conducted by [73] to better understand RNA interference activity in *C2C12* myoblasts, new genes involved in myogenic differentiation were identified. One of the genes identified through Gene Ontology annotation analysis was *BRIX1*, which was also identified as active in the process of the development and deposition of muscle tissue.

Among the CCs enriched for REA, the large ribosomal subunit stands out. Ribosomes are divided into two subunits: the small subunit and the large subunit. Each subunit has distinct ribosomal RNAs and associated ribosomal proteins (r-proteins); the small subunit is responsible for the interaction between mRNA codons and tRNA anticodons, while the large subunit contains the rRNAs that interact to create the actual peptidyl transferase site within the ribosome [74]. According to [75], the study of ribosomal specialization is an emerging topic that highlights an absolutely new concept explaining the regulation of gene expression. Thus, finding such a CC in this study reinforces the thesis proposed by [76] that ribosomes are an important regulator of the growth and maintenance of skeletal muscle, altering cells’ migratory capacity.

Finally, enrichment allowed for the identification of some MFs. An important function potentially associated with the REA that should be mentioned was GTPase regulator activity. GTPases, also known as guanine nucleotide binding proteins (G proteins), are a large family of proteins that control many processes in the cell, such as cell communication, vesicle movement, and control of cell shape and movement. Rho Guanosine triphosphate hydrolases, for example, are molecular switches that oscillate between an inactive state with guanosine diphosphate (GDP) and an active state with guanosine triphosphate (GTP) during signal transduction. As a result, they control various cellular and physiological processes [77]. Several studies report that Rho GTPases play a crucial role during muscle development, regeneration, and function [78,79,80]. According to [81], the complete understanding of all biological programs regulated by Rho GTPase may be of interest not only for the basic understanding of skeletal muscle biology, but also as a way to develop new therapies for diseases associated with skeletal muscle. Therefore, this can also be an alternative to understand how this MF can influence the deposition of muscle in the carcass through the measurement of the REA.

With regard to the SFT characteristic, it is worth highlighting its great importance for the meat industry and for the quality of meat, in addition to promoting sensory improvements in meat products. This characteristic promotes the improvement of the production process, since its adequate proportion can avoid condemnation of the carcass due to the cold shortening factor, wherein carcass temperature drops quickly and muscle pH remains high as a result of meat toughening. Another recurring factor in the lack of subcutaneous fat is the burning of the carcass due to low temperatures in refrigerating chambers. Therefore, this feature is important for the study we conducted [82,83,84]. Enrichment analysis allowed us to identify several pathways; however, two of them stood out for their relationship with the studied phenotype. The first was the pathway of the digestion and absorption of fats. Fat is an important source of energy from food and the correct metabolization of fatty acids is fundamental for the maintenance of the organism. However, when caloric balance is positive, fat is deposited in the organs and tissues, including the subcutaneous region, which suggests that this route may indirectly act in the deposition of lipids, altering the SFT of a carcass [85].

A study conducted by [86], using Hanwoo animals, also identified candidate genes involved in the yield of cuts when performing KEGG enrichment for the bta04975 pathway. One gene that was enriched for the pathway of fat digestion and absorption was the mitochondrial triglyceride transfer protein *MTTP* gene. This gene was first identified as a protein found in the ER, capable of assisting in the transfer of neutral lipids to nascent Apolipoprotein B (APOB) [87]. According to [88], although APOB-containing lipoproteins are crucial for lipid transport, their excess and high concentrations of triglycerides and cholesterol in humans generate various metabolic diseases such as obesity. One of the characteristics of adiposity is the deposition of fat in subcutaneous tissues, which can be related to evidence from this study obtained by the enrichment of pathways for SFT.

Another relevant pathway identified in this study for SFT was the insulin-signaling pathway. Insulin is the best known anabolic hormone and is essential for the maintenance of glucose homeostasis and cell growth and differentiation. This hormone is capable of stimulating lipogenesis in the liver and adipocytes and reducing lipolysis [89]. With the help of insulin, glucose is converted to fatty acids, circulated in other regions of the organism and stored as fat in adipose tissues, as well as in the subcutaneous region. In addition, a factor called insulin resistance may be associated with the studied SFT phenotype. Ref. [90], conducting a study to evaluate insulin resistance during the days of confinement in *Bos indicus* beef cattle receiving a finishing diet with a high concentrate content, demonstrated that by increasing the days of confinement, insulin resistance also increased, showing that it may be linked to the accumulation of adipose tissue and weight gain. SFT is desirable in the correct proportions; however, its excess can represent a financial loss for the producer when animals are marketed by yield, because in the slaughter line, before weighing the carcass, excess fat is removed, and consequently there is a reduction in carcass weight [91].

Important biological processes for SFT were identified, with emphasis on three, the first being the negative regulation of the lipid biosynthetic process. Lipids are organic molecules formed by the bond between fatty acids (FAs) and alcohol; that is, the biosynthesis of lipids depends on numerous reactions which originate FAs, which are the forming units of these molecules [92]. The biosynthesis of fatty acids mainly takes place in the liver, in adipose tissue, and in active mammary glands through a multienzyme system present in the cytosol of animal cells, known as the fatty acid synthetase complex (FAS complex). This process is directly associated with the deposition of lipids in adipose tissue, which may contribute to the SFT characteristic. White adipose tissue, found in mammals, is distributed in several deposits in the body, anatomically classified as subcutaneous adipose tissue (SAT) and visceral adipose tissue (VAT). SAT is mainly represented by deposits under the skin in the abdominal, gluteal, and femoral regions, and SFT evaluation is a tool to verify it. However, the BP found in this study has a regulatory function, and this can be explained by the gene enriched for this process, insulin-induced gene 1 (*INSIG1*). As previously mentioned, insulin is a hormone capable of breaking glucose molecules into FAs, and therefore, depending on its participation, the process of depositing fat in subcutaneous tissues can be modulated. In a study conducted by [93], the authors highlighted the participation of brain sensitivity to insulin in the development of body weight and the distribution of fat in the body, which corroborates the information cited here.

Another BP identified in this study is called intermembrane lipid transfer. According to [94], intermembrane lipid transfer is a fundamental process for many cellular functions and therefore crucial for cell life. Moreover, according to the authors, intermembrane lipid transfer is found in all types of lipids, including glycolipids, and is especially critical to the sphingolipid degradation pathway. Sphingolipids are a class of structural lipids in eukaryotic cells that, in addition to constituting the cell membrane, exhibit a cell-signaling function to modulate insulin sensitivity, differentiation, and apoptosis in a tissue-specific manner. Enphigolipids, including ceramides and sphingosine-1-phosphate (*S1P*), regulate many signaling pathways related to the development of adiposity and metabolic syndrome and as a consequence are able to alter subcutaneous fat deposition [95]. The gene enriched for this BP, *MTTP*, is also enriched for the fat digestion and absorption pathway mentioned earlier in this section, which corroborates our analysis because this gene acts in lipid transfer and is also associated with metabolic diseases and adiposity as well as the sphingolipids highlighted here.

Finally, we found MFs relevant to SFT; however, one of them deserves to be highlighted due to its association with this specific characteristic, namely lipid transfer activity. Lipids are transported between different cellular compartments or even between different body particles and thus play a fundamental role in maintaining cell membrane structure, energy storage, and the regulation of various pathways [96,97]. Correct fat deposition is strictly dependent on lipid metabolism in general [98,99]. As previously mentioned, an important example of lipids are triglycerides. They are transported from adipose tissue to muscles; thus, they are used as an energy source in a process involving the action of transport proteins that remove triglycerides from fat cells and transport them to muscle cells. A dysregulation of lipid transfer may also be associated with metabolic disorders and adiposity, as well as the other lipid-dependent pathways cited above [100]. In addition, the *MTTP* gene also appears as an enriched gene for this pathway, which suggests its strong association with lipid metabolism and, consequently, with the characteristic of SFT. A study conducted by [54] with the aim of characterizing essential molecules related to feed efficiency in different breeds of beef cattle identified the *MTTP* gene as fundamental for lipid metabolism. This gene is involved in the regulation of lipid homeostasis and serum lipid levels [101]. According to [102], the deletion of this gene results in the inability to secrete, and much lower levels of, low-density triglyceride lipoproteins. Such discoveries show us the importance of lipid metabolism and the ways in which these molecules act in the regulation of fat deposition and consequently in altering SFT in animals. 

As for the SF7 phenotype, the enrichment of KEGG pathways helped us identify numerous pathways among which those most related to this specific characteristic stood out. The first pathway was the biosynthesis of unsaturated fatty acids. FAs are carboxylic acids classified according to the length of their carbon chains, the presence or absence of double bonds, and the configuration of the hydrogen atom [103]. In beef, the main categories of fatty acids are saturated fatty acids (SFAs, approximately 46% of total lean FAs), which do not have double bonds, monounsaturated fatty acids (MUFAs, approximately 46% of total lean fatty acids), which do have double bonds, and polyunsaturated fatty acids (PUFAs, approximately 7% of total lean FAs), with two or more double bonds [104]. The ingestion of unsaturated fatty acids is beneficial for consumers to a certain extent and is often indicated for consumption, which corroborates research that aims to change the fatty acid profile of beef, making this product healthier [105]. In addition, UFAs directly influence the tenderness of meat, because, among other factors, they have a low melting point, which makes the digestibility of meat more pleasant. For [106], the effect of FAs on the tenderness of meat products is due to the different melting points of FAs in meat; for example, in the 18C group, stearic acid (18:0) melts at 69.6 °C, oleic acid (18:1) at 13.4 °C, 18:2 at −5 °C, and 18:3 at −11 °C, so that as unsaturation increases, the melting point decreases. Thus, the aforementioned route can contribute to SF7 by positively influencing the tenderness of the meat.

The second pathway enriched for SF7 follows the same topic as the first. The pathway highlighted here is fatty acid metabolism. However, this time the pathway is not restricted to the metabolism of just one type of fatty acid, but to all types in general. Fatty acids are fundamental components of fats and oils present in the body and in food. They are organic molecules that vary in length and saturation, referring to the amount of double bonds between the carbon atoms in the chain. Based on this, FAs can be categorized into three main types: saturated, unsaturated, and trans FAs [107,108]. The quality of the FAs consumed can have a significant impact on the health of consumers in general, which is linked to the research area involving the meat quality called the fatty acid profile (FAP) [105]. In addition to having an impact on consumers’ health, the FAP may be associated with meat tenderness, measured in this study through SF, which is the focus of this section. For both KEGG pathways cited in this section, the enriched gene was stearoyl-CoA desaturase 5 (*SCD5*). *SCD5* is one of the genes in the groups that encode stearoyl-coenzyme A desaturase, a protein present in the membrane of the endoplasmic reticulum and which is associated with increased fat accumulation and responsible for catalyzing the conversion of saturated fat into monounsaturated fatty acids in various body tissues [109]. In a study prepared by [110] to evaluate the carcass and meat quality characteristics of Nellore females, the author reported the relevance of this gene in lipid biosynthesis and highlighted the importance of working with *SCD5* to improve the quality and accumulation of fat in beef. With an increase in intramuscular fat deposition, there is an increase in meat tenderness, one of the reasons being that fat helps lubricate mastication, tending to dilute the connective tissue content of meat [111]. This corroborates the enrichment of the *SDC5* gene enriched for the SF7 characteristic discussed in this section.

Through the enrichment of pathways, we found significant BPs affecting SF. The most relevant of these for this phenotype is the development of the mesoderm. The specific result of this process is the progression of the mesoderm over time, from its formation to its mature structure. Skeletal muscle originates in the mesoderm layer of the embryo during its development, at which point myoblasts migrate, proliferate, and fuse to form myotubes, which mature by further fusion of myofibrils to form muscle fibers. This premise allows us to understand the importance of the mesoderm for the development of muscle tissue. In addition to the development of skeletal muscle, the mesoderm is responsible for the development of cartilage, blood, and connective tissue [112]. In muscles, connective tissue exists at three hierarchical levels, which are independent but interrelated and which are constituents of the extracellular matrix, called the epimysium, perimysium, and endomysium [113]. In a study conducted by [114], the authors reported a greater sensitivity to tenderness and juiciness of meat slices when meat with less connective tissue was provided to tasters in sensory panels. In addition, the more connective tissue the meat contained, the more it was detected by tasters. This demonstrates that the BPs discussed here can have a direct effect on connective tissue deposition, changing the perception of the resulting product’s tenderness.

In the case of CCs, through the enrichment of pathways, we identified a component fundamentally relevant to SF: the sarcoplasmic reticulum (SR). The SR is a structure found in muscle cells, especially in striated muscle, which encompasses skeletal and cardiac muscle, and plays a key role in regulating muscle contraction [115]. Skeletal muscle is the tissue that makes up commercialized meat cuts, and so the SR is a determining factor for the phenotype discussed here. The SR is responsible for storing calcium ions in muscle cells, and during contraction, an electrical impulse penetrates the muscle fibers through T tubules, where they transmit the electrical stimulus to the SR, and as a consequence, cause the release of calcium into the cytoplasm. Calcium released into the SR is rapidly imported across the outer mitochondrial membrane via voltage-gated anion channels. What follows is a series of events that cause muscles to contract. Finally, when calcium is removed from the cytoplasm back to the SR, muscle contraction ceases [116,117]. A work proposed by [118] concluded that the inhibition of mitochondrial calcium uptake promotes an increase in the concentration of calcium in the cytosol in bovine longissimus thoracis et lumborum muscle, and that the inhibition of mitochondrial calcium uptake causes positive impacts on autolysis, calpain-1, on calpastatin degradation, meat tenderness and texture properties, and protein degradation. This confirms the characteristic evaluated in this study and the importance of the enriched endoplasmic reticulum component.

Finally, the enrichment of pathways allowed us to obtain several important MFs; however, the protein-binding scaffold stands out here. Structure proteins play a fundamental role in the coordination of signal transduction cascades [119]. Anchored protein kinase A is an example of a scaffold protein that acts as a platform for protein kinase A (PKA)-signaling pathway enzymes to link to other molecular pairs so that these enzymes can communicate and perform functions in a coordinated manner. A study conducted by [120] demonstrated that phosphorylation is related to meat tenderization during the postmortem period, and that incubation with PKA was effective in altering the state of phosphorylation of proteins. Furthermore, ref. [121] indicated that PKA has numerous targets within the actin filament when exposing the entire fiber to the catalytic subunit of PKA. This demonstrates the importance of studies involving PKA and its influence on meat tenderness. Understanding the protein-binding scaffold MF can be a viable tool for this purpose.

The IMF characteristic, also called marbling fat, is a highly desired characteristic in beef, as it contributes to the juiciness, tenderness, and flavor of the meat, adding value to the final product and promoting the slaughterhouse industry. Therefore, in-depth investigations on the metabolic processes that affect this characteristic are necessary, and one of the tools for this purpose is the enrichment of pathways. The enrichment of KEGG pathways allowed us to identify some important pathways associated with IMF; among them, one that stands out in particular for its relevance is the ABC transporter pathway. The ABC transport system is a group of active transport systems found in the cell membranes of living organisms that plays a key role in the transport of various molecules, such as nutrients, toxins, and ions, among other substances [122,123]. It is believed that the genes belonging to this group, called the ABC superfamily, participate in the absorption and secretion of endogenous and exogenous substances and in muscle regeneration [124]. A study conducted by [125] evaluating the profile of DNA methylation and its interference in the tenderness of meat from Angus cattle (*Bos taurus*) identified 21 members of the ABC family which are differently methylated between tough and tender meats. According to [126], the different methylation of these ABC transporters can influence lipid transport, interfering with the metabolism of FAs in bovine *longissimus lumborum* muscles. In our work, we identified two genes from this family being enriched for the ABC transport pathway that belonged to the aforementioned superfamily. They were *ABCC11* and *ABCC12*. This agrees with the works mentioned, as these genes can interfere with lipid dynamics, influencing the deposition of intramuscular fat in meat.

Among the different BPs identified in our study, two stood out for having a close relationship with IMF, the first being lipid oxidation. Lipid oxidation is a chemical process in which lipids react with atmospheric oxygen or with reactive oxygen species, and as a consequence, changes occur in their molecular structures. Such a process can occur in food, cell membranes, and biological tissues [127]. According to Scollan and colleagues, an excessive increase in lipid oxidation during long maturation periods can lead to a loss of nutritional value and product acceptability [104]. The phenotype studied in this section concerns the deposition of fat in the intramuscular region. This characteristic is desired by most consumers; however, the presence of this fat can contribute to lipid oxidation, causing harm to the meat during maturation. According to [128], three main points need to be taken into account in the response to intramuscular fat oxidation: the content of polyunsaturated FAs, the amount of reactive oxygen species produced, and the level of natural antioxidants in the meat. In beef, there are natural antioxidants, such as α-tocopherol, which help with the natural control of meat oxidation. Tocopherol levels can be altered by adjusting the animals’ diet [129]. Therefore, the BP identified in our study can contribute to works that aim to minimize oxidative effects in meat, providing important information for understanding the predisposition to lipid oxidation of the product.

The second important BP enriched for IMF is lipid modification. Lipid modifications are chemical changes that occur in lipids, which are organic molecules that play a fundamental role in several BPs. Such modifications can occur naturally or can be induced by a BP, whether chemical or enzymatic. This can significantly impact the structural, biological, and functional properties of lipids [130]. Lipid oxidation, mentioned earlier, is an example of this process, which in this case involves the modification of lipids through reactions with oxygen or its reactive species. Therefore, the two BPs enriched for IMF in this work complement each other in terms of information. It is important to point out that in both BPs, the enriched gene was ion peptidase 2, peroxisomal (*LONP2*). There is evidence that *LONP2* plays a role in peroxisome morphology and degradation [131,132]. Peroxisomes are cytoplasmic organelles that contain digestive enzymes responsible for the degradation of fats and amino acids within cells [133,134]. This information complements what was mentioned here about lipid modifications, as this gene is related to the beta-oxidation of some FAs, components which form lipids and which can compromise the deposition of IMF. This information can promote knowledge about the behavior of lipid metabolism as a result of fat deposition in the intramuscular portion of meat.

With regard to CCs identified for IMF, one of them in particular stood out in terms of complementing the information discussed so far; the component in question are peroxisomes. Peroxisomes perform crucial functions for cellular metabolism, such as lipid metabolism and long-chain fatty acid (LCFA) oxidation [135]. LCFAs are crucial for energy metabolism. Among the most abundant LCFAs are oleic acid, linoleic acid, and arachidonic acid [136,137]. A study carried out by [31], investigating the deposition and composition of marbling fat in Nellore cattle using the GWAS tool, demonstrated that oleic acid (C18:1 cis-9) was the most abundant FA detected. Therefore, understanding the participation of peroxisomes in the oxidation of these FAs may be essential in order to understand the impact of this component on the deposition of IMF in Nellore cattle.

Finally, pathway enrichment allowed us to find an MF associated with IMF that stood out among the others: the gated channel activity MF. Ion channels are characterized by being transmembrane proteins, forming pores in cell membranes, and allowing the selective passage of ions such as calcium (Ca^2+^), sodium (Na^+^), and potassium (K^+^). In the case of closed channels, we refer to the inactive state in which these channels are found and in which they do not allow for the entry of certain ions [138,139]. This activity is mediated by electrical voltage on the membrane, by the binding of regulatory molecules, or by mechanical factors. Intramuscular fat deposition is dependent on lipogenesis, which in turn is regulated by the transmembrane passage of ions such as Ca^2+^, as these play a role in signaling activating enzymes capable of modulating lipogenesis [140]. As an example, we can mention the hormonal responses capable of increasing Ca^2+^ levels, triggering the activation of enzymes that promote the deposition of FA and triglycerides [141]. Thus, the highlighted MF is capable of influencing lipid homeostasis, being able to change the proportions of IMF deposited in the meat, thus compromising its quality.

## 5. Conclusions

The results obtained suggest a multifactorial genomic locus associated with the REA, SFT, SF7, and IMF in Nellore beef cattle. Using genome-wide association studies (GWAS), we found nine genomic regions associated with the REA, eight regions associated with SFT, four regions associated with SF, and three genomic regions associated with IMF. The MeSH terms heat stress disorders and life cycle stages were related to the **REA**, insulin and nonesterified fatty acids were associated with **SFT**, apoptosis and HSP27 heat shock proteins were associated with **SF7**, and finally, matrix metalloproteinase 2 was related to **IMF**. These genes were enriched for metabolic pathways, biological processes, cellular components, and molecular functions mainly related to differentiation, muscle cell proliferation, ribosomal metabolism, and lipid metabolism, such as the fatty acid metabolism pathway, ribosome pathway, and lipid biosynthetic processes. For both traits analyzed, we identified genes involved in biological processes that can positively or negatively regulate lipid biosynthesis, muscle growth, and adipogenesis in cattle. In addition, our study provided important information on the genomic regions involved in the analyzed traits, which can contribute to improving beef quality.

## Figures and Tables

**Table 1:** Descriptive statistics, variance components, and genomic heritability.

Trait	N	CG	Mean	Min	Max	$\sigma_{a}^{2}$	$\sigma_{a}^{2}$	$h^{2}\pm SD$	NAG	$\sigma_{a}^{2}g$	$\sigma_{e}^{2}g$	$h^{2}g\pm SD$
REA	2417	45	46.85	18.59	122.88	3.84	32.65	0.11 ± 0.05	2181	5.12	31.64	0.14 ± 0.03
SFT	2368	45	3.53	0.20	10.28	0.64	2.56	0.20 ± 0.07	2181	0.39	2.81	0.12 ± 0.03
SF7	2431	45	6.64	1.43	13.98	0.74	3.33	0.18 ± 0.08	2181	0.41	3.65	0.10 ± 0.04
IMF	1854	33	1.76	0.01	4.52	0.12	0.47	0.21 ± 0.09	2181	0.13	0.47	0.21 ± 0.05

N: number of animals; CG: contemporaneous group number; Min: minimum; Max: maximum; $\sigma_{a}^{2}$: additive genetic variance; $\sigma_{e}^{2}$: residual variance; *h*^2^ ± *SD*: estimate of heritability and standard deviation; NAG: number of animals genotyped; $\sigma_{a}^{2}g$: additive genetic variance with genome; $\sigma_{e}^{2}g$: residual variance with genome; and $h^{2}g\pm SD:$ estimate of heritability and standard deviation with genome.

**Table 2:** Characterization of 1 Mb genomic windows explaining 0.5% or more of the genetic variance for meat and carcass quality traits.

Trait	BTA_Mb	Genomic Window (First–Last SNP)	#SNPs	%Var
REA	1_139	rs135422759–rs43160114	318	0.58
	1_143	rs108998682–rs110047615	225	0.51
	2_103	rs133661878–rs110353021	179	0.71
	8_59	rs135591081–rs134301958	206	0.63
	9_27	rs133075840–rs135200101	319	0.58
	11_4	rs41632961–rs110740485	250	0.59
	11_85	rs136279193–rs133538333	245	0.56
	16_67	rs135309995–rs41617334	257	0.73
	17_67	rs133453006–rs109252298	202	0.50
	20_39	rs133317890–rs135526251	295	0.51
SFT	1_155	rs42869710–rs109687330	276	0.54
	2_105	rs134465164–rs132698232	220	1.57
	4_117	rs110440609–rs134013499	178	1.01
	6_26	rs109588250–rs43453787	275	0.72
	6_78	rs43057211–rs109068276	303	1.61
	11_95	rs110260566–rs109229702	190	0.62
	12_2	rs135898108–rs137788754	362	0.52
	12_39	rs133691144–rs109792923	184	0.54
	12_41	rs136100155–rs109234911	162	0.57
	17_47	rs133188023–rs110546764	323	0.71
	24_31	rs109644776–rs109246799	270	0.57
SF7	2_57	rs43312105–rs135234804	253	1.02
	5_68	rs133020210–rs132713848	210	0.77
	6_99	rs135511664–rs134451951	228	0.58
	11_28	rs43677070–rs136376171	257	0.54
	24_48	rs136072970–rs134381469	204	0.51
	27_7	rs134965123–rs42619553	266	0.90
IMF	6_68	rs135331134–rs41653747	303	0.76
	7_59	rs109901778–rs133094202	228	0.54
	8_103	rs134125299–rs136759870	258	0.71
	17_9	rs42655606–rs135933217	290	0.57
	18_16	rs109942482–rs133384244	264	0.56
	24_61	rs133529820–rs135629046	268	0.62
	29_12	rs42670744–rs133896635	189	0.54

BTA_Mb = position based on the genome of Bos_taurus.ARS-UCD1.2.104 (NCBI); Genomic window = SNPs present at the beginning and end of the genomic window; #SNPs = number of SNPs present in the genomic window; %Var = percentage of genetic variance explained by the genomic window.

**Table 3:** Genes distributed in genomic windows explaining more than 0.5% of additive genetic variance for the rib eye area.

Chr	Genomic Range (Start–End Position)	Genes	%Var
1	139678041–140081419	*CPNE4*, *MRPL3*, *NUDT16*, *NEK11*, *RF00026*	0.58
	143048262–144071071	*FAM3B*, *M X2*, *MX1*, *TMPRSS2*, *RIPK4*, *PRDM15*, *C2CD2*, *ZBTB21*, *UMODL1*, *ABCG1*, *TFF3*	0.51
2	103520024–103950562	*ABCA12*, *RF00156*, *ATIC*, *FN1*	0.71
8	59427078–60332994	*RF00410*, *FAM205C*, *PHF24*, *RF00026*, *DNAJB5*, *VCP*, *FANCG*, *PIGO*, *STOML2*, *FAM214B*, *UNC13B*, *RUSC2*, *FAM166B*, *TESK1*, *CD72*, *SIT1*, *RF00030*, *CCDC107*, *ARHGEF39*, *CA9*, *TPM2*, *TLN1*, *CREB3*, *GBA2*, *RGP1*, *MSMP*	0.63
9	26690578–27348055	*RF00026*, *NKAIN2*	0.58
11	3686196–4599212	*INPP4A*, *COA5*, *UNC50*, *MGAT4A*, *RF00425*, *TSGA10*, *C2orf15*, *LIPT1*, *MITD1*, *MRPL30*, *LYG2*, *TXNDC9*, *EIF5B*, *REV1*	0.59
	84872440–85408763	*RF00004*, *TRIB2*, *RF00279*	0.56
16	66960790–68078846	*C16H1orf21*, *EDEM3*, *FAM129A*, *RNF2*, *TRMT1L*, *SWT1*, *IVNS1ABP*	0.73
17	67357452–68355258	-	0.50
20	39073246–39413736	*PRLR*, *AGXT2*, *DNAJC21*, *BRIX1*, *RAD1*, *TTC23L*, *RF00003*, *RAI14*	0.51

Chr = chromosome; %Var = percentage of variance explained by the genomic window.

**Table 4:** Genes distributed in genomic windows that explained more than 0.5% of additive genetic variance for subcutaneous fat thickness.

Chr	Genomic Range (Start–End Position)	Genes	%Var
1	155383458–156185921	*DAZL*, *PLCL2*, *RF00026*, *TBC1D5*	0.54
2	105338358–106321176	*IGFBP2*, *IGFBP5*, *TNP1*	1.57
4	117153120–118274566	*HTR5A*, *PAXIP1*, *INSIG1*, *CNPY1*, *RF00026*, *RBM33*, *SHH*	1.01
6	26167969–27080766	*DAPP1*, *C4orf54*, *MTTP*, *TRMT10A*, *C6H4orf17*, *ADH7*, *ADH6*, *ADH4*, *ADH5*, *METAP1*, *EIF4E*	0.72
	78991885–79313522	*ADGRL3*	1.61
11	95314593–96511560	*NEK6*, *‘PSMB7*, *ADGRD2*, *NR5A1*, *NR6A1*, *bta-mir-181a-2*, *bta-mir-181b-2*, *OLFML2A*, *RF00026*, *WDR38*, *RPL35*, *ARPC5L*, *GOLGA1*, *SCAI*, *RF00264*, *PPP6C*, *RABEPK*, *HSPA5*, *GAPVD1*, *RF00026*, *RF00020*, *MAPKAP1*	0.62
12	1527007–2695362	*TDRD3*, *DIAPH3*	0.52
	39640396–39640466	*RF01161*	0.54
	41873452–42872104	-	0.57
17	47976840–48975164	-	0.71
24	31906526–32905155	-	0.57

Chr = chromosome; %Var = percentage of variance explained by the genomic window.

**Table 5:** Genes distributed in genomic windows that explained more than 0.5% of additive genetic variance for shear force at 7 days’ ageing.

Chr	Genomic Range (Start–End Position)	Genes	%Var
2	56711720–57011911	-	1.02
5	67612447–68813411	*STAB2*, *NT5DC3*, *HSP90B1*, *C5H12orf73*, *TDG*, *GLT8D2*, *HCFC2*, *NFYB*, *TXNRD1*, *CHST11*, *SLC41A2*	0.77
6	99233279–100341274	*SCD5*, *SEC31A*, *THAP9*, *LIN54*, *bta-mir-2447*, *COPS4*, *RF00156*, *PLAC8B*, *PLAC8*, *COQ2*, *HPSE*, *bta-mir-2446*, *MRPS18C*, *ABRAXAS1*, *GPAT3*, *RF00265*	0.58
11	27935104–29210371	*PRKCE*, *EPAS1*, *TMEM247*, *ATP6V1E2*, *RHOQ*, *PIGF*, *CRIPT*, *SOCS5*, *MCFD2*	0.54
24	48278442–49277504	-	0.51
27	7955174–8952985	-	0.90

Chr = chromosome; %Var = percentage of variance explained by the genomic window.

**Table 6:** Genes distributed in genomic windows that explained more than 0.5% of additive genetic variance for intramuscular fat.

Chr	Genomic Range (Start–End Position)	Genes	%Var
6	67925293–69167282	*CORIN*, *NFXL1*, *CNGA1*, *NIPAL1*, *TXK TEC*, *SLAIN2*, *SLC10A4*, *ZAR1*, *FRYL*, *RF00001*, *OCIAD1*	0.76
7	59547160–60543762	*-*	0.54
8	103059951–104010320	*RF00001*, *SUSD1*, *RF00026*, *PTBP3*, *HSDL2*, *KIAA1958*, *INIP*, *SNX30*, *SLC46A2*	0.71
17	9882597–10881624	-	0.57
18	16921890–17921036	*ABCC12*, *ABCC11*, *LONP2*, *SIAH1*	0.56
24	61023178–62021049	*-*	0.62
29	12754057–13751988	*PRCP*, *FAM181B*	0.54

Chr = chromosome; %Var = percentage of variance explained by the genomic window.

## Data Availability

The data presented in this study are available upon request from the corresponding author. The data are not publicly available as they are part of the data collection of the Animal Genetic Improvement, Biotechnology and Transgenics Group (NAP-GMABT) of the Department of Veterinary Medicine of the Faculty of Animal Science and Food Engineering of the University of São Paulo.

## Supplementary Materials

The following supporting information can be downloaded at: https://www.mdpi.com/article/10.3390/metabo14010006/s1.
