# Supplementary material for: Genome-Wide Association (GWAS) Applied to Carcass and Meat Traits of Nellore Cattle
Source: Metabolites. 2023 Dec 21;14(1):6. doi: 10.3390/metabo14010006 (PMC10818672; doi:10.3390/metabo14010006)
Supplement: Supplementary file 1 [file metabolites-14-00006-s001.zip › metabolites-2683569-supplementary.pdf]

Supplementary Archive 1

Table S1 - Enriched MeSH terms (FDR<0.05) related to the Ribeye Area (REA) trait, with their respective candidate genes.

MeSH ID[1]	MeSH Terms	Genes	p-value	FDR[2]
D007315	Insemination, Artificial	FN1, MX2, MX1	2.06E-05	1.38E-03
D000437	Alcoholism	FN1	9.06E-04	3.02E-03
D003316	Corneal Diseases	FN1	9.06E-04	3.02E-03
D018186	Pneumovirus Infections	MX1	9.06E-04	3.02E-03
D004881	Ergotism	PRLR	1.81E-03	4.53E-03
D064547	Myxovirus Resistance Proteins	MX2, MX1	4.62E-05	5.36E-03
D005355	Fibrosis	FN1	2.71E-03	5.43E-03
D012128	Respiratory Distress Syndrome, Adult	FN1	3.62E-03	6.03E-03
D015088	2',5'-Oligoadenylate Synthetase	MX2, MX1	1.21E-04	7.00E-03
D018882	Heat Stress Disorders	PRLR	5.42E-03	7.75E-03
D000249	Adenosine Monophosphate	LIPT1, ATIC	2.29E-04	8.87E-03
D007372	Interferons	MX2, MX1	4.56E-04	1.32E-02
D004717	Endometrium	FN1, MX2, MX1, PRLR	3.70E-04	1.49E-02
D014599	Uterus	MX2, PRLR, MX1	5.93E-04	1.49E-02

D007107	Immune System	MX2, MX1	8.58E-04	1.49E-02
D014452	Ubiquitins	MX2, MX1	1.28E-03	1.84E-02
D016085	Bronchoconstrictor Agents	FN1	1.52E-03	1.84E-02
D016210	Methacholine Chloride	FN1	1.52E-03	1.84E-02
D026122	Factor XIIIa	FN1	1.52E-03	1.84E-02
D019204	GTP-Binding Proteins	FN1, MX2, MX1	1.53E-03	1.84E-02
D011257	Pregnancy Proteins	MX2, MX1, PRLR	1.59E-03	1.84E-02
D013203	Staphylococcal Infections	FN1	2.25E-02	2.81E-02
D011270	Pregnancy, Animal	MX2, MX1, PRLR	9.73E-04	2.86E-02
D008018	Life Cycle Stages	FN1	1.71E-03	2.86E-02
D011999	Recruitment, Neurophysiological	UNC13B	1.71E-03	2.86E-02
D041681	NIH 3T3 Cells	FN1, MX2	2.84E-03	3.69E-02
D007370	Interferon Type I	MX2, MX1	3.96E-03	3.77E-02
D017469	Receptors, Fibronectin	FN1	4.55E-03	3.77E-02
D018829	Adhesins, Bacterial	FN1	4.55E-03	3.77E-02
D053484	RecQ Helicases	VCP	4.55E-03	3.77E-02
D010064	Embryo Implantation	FN1, MX2, MX1	3.12E-03	3.92E-02

D013329	Structure-Activity Relationship	FN1,PRLR, MGAT4A	3.95E-03	3.92E-02
D004705	Endocytosis	FN1, PRLR	4.92E-03	3.92E-02
D002980	Climate	PRLR	5.11E-03	3.92E-02
D014162	Transfection	MX2,MX1, PRLR, MGAT4A	5.26E-03	3.92E-02
D005865	Gestational Age	MX2, MX1	6.97E-03	4.07E-02
D017384	Sequence Deletion	MX1, MGAT4A	8.32E-03	4.07E-02
D001422	Bacterial Adhesion	FN1	8.50E-03	4.07E-02
D017440	Photoperiod	PRLR	8.50E-03	4.07E-02
D031204	Caloric Restriction	LOC516849	8.50E-03	4.07E-02
D012995	Solubility	FN1, MGAT4A	9.78E-03	4.17E-02
D019943	Amino Acid Substitution	MX2, MX1, PRLR	9.97E-03	4.17E-02
D053667	Syndecans	FN1	6.06E-03	4.39E-02
D060749	alpha-2-HS-Glycoprotein	LOC516849	6.06E-03	4.39E-02
D011650	Pulmonary Alveoli	FN1	5.45E-03	4.72E-02
D041702	BALB 3T3 Cells	MX1	5.45E-03	4.72E-02
D003871	Dermatan Sulfate	FN1	7.57E-03	4.88E-02
D007315	Insemination, Artificial	FN1, MX2, MX1	2.06E-05	1.38E-03

Table S2 - Enriched MeSH terms (FDR<0.05) related to the Subcutanous Fat Thickness (SFT) trait, with their respective candidate genes.

MeSH ID[1]	MeSH Terms	Genes	p-value	FDR[2]
D042783	Endothelial Cells	HSP90B1, HPSE, TXNRD1, PRKCE, LOC505479	6.32E-05	1.89E-03
D017209	Apoptosis	TXNRD1, PRKCE, TDG, LOC505479	5.01E-05	2.00E-03
D055551	HSP27 Heat-Shock Proteins	HSP90B1, HPSE	5.48E-05	5.21E-03
D000532	Altitude Sickness	EPAS1	1.13E-03	5.66E-03
D006976	Hypertension, Pulmonary	EPAS1	9.03E-03	2.26E-02
D006052	Gold Sodium Thiomalate	TXNRD1	1.03E-03	2.45E-02
D018501	Antirheumatic Agents	TXNRD1	1.03E-03	2.45E-02
D051767	Early Growth Response Protein 2	SCD5	1.03E-03	2.45E-02
D006943	Hyperglycemia	HPSE	1.80E-02	2.67E-02
D016908	Gram-Positive Bacterial Infections	MIR2285AC	2.13E-02	2.67E-02
D054468	Axoneme	PRKCE	2.52E-03	2.68E-02
D004730	Endothelium, Vascular	EPAS1, TXNRD1, PRKCE	2.68E-03	2.68E-02
D014777	Virus Diseases	MIR2446, MIR2447	3.07E-02	3.07E-02
D004847	Epithelial Cells	PRKCE, TDG, MIR2285AC	5.67E-03	3.23E-02
D001940	Breast	HSP90B1	7.55E-03	3.23E-02

D001980	Bronchi	PRKCE	7.55E-03	3.23E-02
D064448	Filamins	HPSE	7.55E-03	3.23E-02
D054481	Thioredoxin Reductase1	TXNRD1	2.06E-03	3.27E-02
D058473	Receptors, Purinergic P2Y2	HPSE	2.06E-03	3.27E-02
D044502	Thymine DNA Glycosylase	TDG	3.09E-03	3.56E-02
D003487	Cyanoacrylates	HSP90B1	4.12E-03	3.56E-02
D010171	Palmitoyl Coenzyme A	SCD5	4.12E-03	3.56E-02
D013880	Thioredoxin-Disulfide Reductase	TXNRD1	4.12E-03	3.56E-02
D053667	Syndecans	HPSE	4.12E-03	3.56E-02
D026361	Reactive Nitrogen Species	PRKCE	5.15E-03	3.67E-02
D051547	Heme Oxygenase-1	TXNRD1	5.15E-03	3.67E-02
D008054	Lipid Peroxides	TXNRD1	6.18E-03	3.67E-02
D018048	Receptors, Purinergic P2	HPSE	6.18E-03	3.67E-02
D064448	Filamins	HPSE	6.18E-03	3.67E-02
D051793	Hypoxia-Inducible Factor 1	EPAS1	7.20E-03	4.03E-02
D000431	Ethanol	PRKCE	8.23E-03	4.07E-02
D008071	Lipoprotein Lipase	TDG	9.25E-03	4.07E-02

D014166	Transferases	STAB2	9.25E-03	4.07E-02
D051745	Protein Kinase C-delta	PRKCE	9.25E-03	4.07E-02
D000001	Calcimycin	PRKCE	1.03E-02	4.07E-02
D011496	Protein Methyltransferases	LOC505479	1.03E-02	4.07E-02
D051795	Hypoxia-Inducible Factor 1, alpha Subunit	EPAS1	1.03E-02	4.07E-02
D004224	Diterpenes	LOC505479	1.13E-02	4.13E-02
D005966	Glucuronidase	HPSE	1.13E-02	4.13E-02
D002614	Chelating Agents	PRKCE	1.23E-02	4.33E-02
D000214	Acyl Coenzyme A	SCD5	1.33E-02	4.53E-02
D018960	Hyaluronan Receptors	STAB2	1.44E-02	4.70E-02
D022502	Stress Fibers	HPSE	1.25E-02	4.71E-02
D032383	Myocytes, Cardiac	HPSE	1.63E-02	4.95E-02
D003593	Cytoplasm	HSP90B1, MRPS18C	1.65E-02	4.95E-02

[1]MeSH ID: MeSH terms identifier; [2]FDR: False Discovery Rate.

Table S3. Enriched MeSH terms (FDR<0.05) related to the Shear Force at 7 Days ageing (SF7) trait, with their respective candidate genes.

**MeSH ID**[1]	MeSH Terms	Genes	p-value	FDR[2]
D000532	Altitude Sickness	EPAS1	1.13E-03	5.66E-03
D002373	Cyclic AMP Receptor Protein	CNGA1	4.25E-04	5.81E-03
D008672	Metals, Alkali	CNGA1	4.25E-04	5.81E-03
D064428	Hyperpolarization-Activated Cyclic Nucleotide-Gated Channels	CNGA1	4.25E-04	5.81E-03
D002414	Cations, Monovalent	CNGA1	8.50E-04	8.71E-03
D002586	Cesium	CNGA1	1.70E-03	1.39E-02
D017956	Photoreceptor Cells, Invertebrate	CNGA1	1.02E-03	1.63E-02
D009712	Nucleotides, Cyclic	CNGA1	2.97E-03	1.74E-02
D011422	Propionates	CNGA1	2.97E-03	1.74E-02
D013439	Sulfhydryl Reagents	CNGA1	4.24E-03	2.17E-02
D006976	Hypertension, Pulmonary	EPAS1	9.03E-03	2.26E-02
D013388	Succinimides	CNGA1	5.51E-03	2.28E-02
D001616	beta-Galactosidase	CNGA1	6.36E-03	2.28E-02
D004527	Egg Proteins	ZAR1	6.36E-03	2.28E-02
D001216	Asparagine	CNGA1	6.78E-03	2.28E-02
D054815	Cyclic Nucleotide-Gated Cation Channels	CNGA1	7.63E-03	2.28E-02

D015221	Potassium Channels	CNGA1	8.47E-03	2.28E-02
D055430	Bone Morphogenetic Protein 15	ZAR1	8.47E-03	2.28E-02
D055429	Growth Differentiation Factor 9	ZAR1	8.89E-03	2.28E-02
D008164	Luminescent Proteins	CNGA1	9.74E-03	2.35E-02
D020778	Matrix Metalloproteinase 2	CNGA1	1.14E-02	2.60E-02
D006943	Hyperglycemia	HPSE	1.80E-02	2.67E-02
D016908	Gram-Positive Bacterial Infections	MIR2285AC	2.13E-02	2.67E-02
D020780	Matrix Metalloproteinase 9	CNGA1	1.35E-02	2.80E-02
D013912	Threonine	CNGA1	1.39E-02	2.80E-02
D012964	Sodium	CNGA1	1.44E-02	2.80E-02
D014777	Virus Diseases	MIR2446, MIR2447	3.07E-02	3.07E-02
D006152	Cyclic GMP	CNGA1	1.86E-02	3.38E-02
D001426	Bacterial Proteins	CNGA1	1.90E-02	3.38E-02
D050505	Mutant Proteins	CNGA1	1.98E-02	3.38E-02
D009068	Movement	CNGA1	2.54E-03	4.07E-02
D055724	Electrophysiological Phenomena	CNGA1	2.54E-03	4.07E-02
D002147	Calmodulin	CNGA1	2.56E-02	4.11E-02
D003432	Cross-Linking Reagents	CNGA1	2.61E-02	4.11E-02

[1]MeSH ID: MeSH terms identifier; [2]FDR: False Discovery Rate.

Table S4. Enriched MeSH terms (FDR<0.05) related to the Intramuscular Fat (IMF) trait, with their respective candidate genes.

MeSH ID[1]	MeSH Terms	Genes	p-value	FDR[2]
D000532	Altitude Sickness	EPAS1	1.13E-03	5.66E-03
D002373	Cyclic AMP Receptor Protein	CNGA1	4.25E-04	5.81E-03
D008672	Metals, Alkali	CNGA1	4.25E-04	5.81E-03
D064428	Hyperpolarization-Activated Cyclic Nucleotide-Gated Channels	CNGA1	4.25E-04	5.81E-03
D002414	Cations, Monovalent	CNGA1	8.50E-04	8.71E-03
D002586	Cesium	CNGA1	1.70E-03	1.39E-02
D017956	Photoreceptor Cells, Invertebrate	CNGA1	1.02E-03	1.63E-02
D009712	Nucleotides, Cyclic	CNGA1	2.97E-03	1.74E-02
D011422	Propionates	CNGA1	2.97E-03	1.74E-02
D013439	Sulfhydryl Reagents	CNGA1	4.24E-03	2.17E-02
D006976	Hypertension, Pulmonary	EPAS1	9.03E-03	2.26E-02
D013388	Succinimides	CNGA1	5.51E-03	2.28E-02
D001616	beta-Galactosidase	CNGA1	6.36E-03	2.28E-02
D004527	Egg Proteins	ZAR1	6.36E-03	2.28E-02

D001216	Asparagine	CNGA1	6.78E-03	2.28E-02
D054815	Cyclic Nucleotide-Gated Cation Channels	CNGA1	7.63E-03	2.28E-02
D015221	Potassium Channels	CNGA1	8.47E-03	2.28E-02
D055430	Bone Morphogenetic Protein 15	ZAR1	8.47E-03	2.28E-02
D055429	Growth Differentiation Factor 9	ZAR1	8.89E-03	2.28E-02
D008164	Luminescent Proteins	CNGA1	9.74E-03	2.35E-02
D020778	Matrix Metalloproteinase 2	CNGA1	1.14E-02	2.60E-02
D006943	Hyperglycemia	HPSE	1.80E-02	2.67E-02
D016908	Gram-Positive Bacterial Infections	MIR2285AC	2.13E-02	2.67E-02
D020780	Matrix Metalloproteinase 9	CNGA1	1.35E-02	2.80E-02
D013912	Threonine	CNGA1	1.39E-02	2.80E-02
D012964	Sodium	CNGA1	1.44E-02	2.80E-02
D014777	Virus Diseases	MIR2446, MIR2447	3.07E-02	3.07E-02
D006152	Cyclic GMP	CNGA1	1.86E-02	3.38E-02
D001426	Bacterial Proteins	CNGA1	1.90E-02	3.38E-02
D050505	Mutant Proteins	CNGA1	1.98E-02	3.38E-02
D009068	Movement	CNGA1	2.54E-03	4.07E-02

D055724	Electrophysiological Phenomena	CNGA1	2.54E-03	4.07E-02
D002147	Calmodulin	CNGA1	2.56E-02	4.11E-02
D003432	Cross-Linking Reagents	CNGA1	2.61E-02	4.11E-02

[1]MeSH ID: MeSH terms identifier; [2]FDR: False Discovery Rate.

Supplementary Archive 2

Table S5 – KEGG functional enrichment related to the Ribeye Area (REA) trait.

ID¹	Description	GeneRatio	BgRatio	p-value	p.adjust	q-value	Gene ID	Count
bta03460	Fanconi anemia pathway	2/30	52/9308	1,20E+14	1,20E+14	4,48E+14	615352/616986	2
bta02010	ABC transporters	2/30	68/9308	2,01E+14	2,01E+14	4,48E+14	523479/510745	2
bta03250	Viral life cycle - HIV-1	2/30	68/9308	2,01E+14	2,01E+14	4,48E+14	280873/280872	2
bta05164	Influenza A	3/30	188/9308	2,21E+14	2,21E+14	4,48E+14	280873/280872/511037	3
bta05165	Human papillomavirus infection	4/30	352/9308	2,53E+14	2,53E+14	4,48E+14	280794/281715/280873/280872	4
bta05215	Prostate cancer	2/30	98/9308	3,94E+14	3,94E+14	4,56E+14	281715/511037	2
bta00670	One carbon pool by folate	1/30	18/9308	5,65E+14	5,65E+14	4,56E+14	506343	1
bta00910	Nitrogen metabolism	1/30	18/9308	5,65E+14	5,65E+14	4,56E+14	511923	1
bta05171	Coronavirus disease - COVID-19	3/30	282/9308	6,12E+14	6,12E+14	4,56E+14	280873/280872/511037	3
bta00230	Purine metabolism	2/30	133/9308	6,80E+14	6,80E+14	4,56E+14	506343/512320	2
bta00511	Other glycan degradation	1/30	23/9308	7,16E+14	7,16E+14	4,56E+14	100139170	1
bta00563	Glycosylphosphatidylinositol (GPI)-anchor biosynthesis	1/30	27/9308	8,36E+14	8,36E+14	4,56E+14	783989	1
bta04261	Adrenergic signaling in cardiomyocytes	2/30	154/9308	8,76E+14	8,76E+14	4,56E+14	497015/281715	2
bta05162	Measles	2/30	160/9308	9,34E+14	9,34E+14	4,56E+14	280873/280872	2
bta03010	Ribosome	2/30	168/9308	1,01E+14	1,01E+14	4,56E+14	614151/614906	2
bta05160	Hepatitis C	2/30	172/9308	1,06E+14	1,06E+14	4,56E+14	280873/280872	2
bta04141	Protein processing in endoplasmic reticulum	2/30	175/9308	1,09E+14	1,09E+14	4,56E+14	519058/507345	2
bta00250	Alanine, aspartate and glutamate metabolism	1/30	38/9308	1,16E+14	1,16E+14	4,56E+14	521553	1
bta04151	PI3K-Akt signaling pathway	3/30	379/9308	1,21E+14	1,21E+14	4,56E+14	280794/281715/281422	3
bta00260	Glycine, serine and threonine metabolism	1/30	45/9308	1,36E+14	1,36E+14	4,56E+14	521553	1
bta00513	Various types of N-glycan biosynthesis	1/30	46/9308	1,38E+13	1,38E+13	4,56E+14	282276	1
bta04510	Focal adhesion	2/30	203/9308	1,39E+14	1,39E+14	4,56E+14	280794/783470	2
bta05030	Cocaine addiction	1/30	48/9308	1,44E+14	1,44E+14	4,56E+14	281715	1
bta04962	Vasopressin-regulated water reabsorption	1/30	49/9308	1,47E+14	1,47E+14	4,56E+14	281715	1
bta01523	Antifolate resistance	1/30	50/9308	1,49E+14	1,49E+14	4,56E+14	506343	1
bta00280	Valine, leucine and isoleucine degradation	1/30	51/9308	1,52E+13	1,52E+13	4,56E+14	521553	1
bta00270	Cysteine and methionine metabolism	1/30	53/9308	1,58E+14	1,58E+14	4,56E+14	521553	1
bta00510	N-Glycan biosynthesis	1/30	55/9308	1,63E+14	1,63E+14	4,56E+14	282276	1
bta00600	Sphingolipid metabolism	1/30	57/9308	1,69E+14	1,69E+14	4,56E+14	100139170	1
bta05134	Legionellosis	1/30	59/9308	1,74E+13	1,74E+13	4,56E+14	507345	1
bta05166	Human T-cell leukemia virus 1 infection	2/30	238/9308	1,78E+14	1,78E+14	4,56E+14	783470/281715	2
bta04714	Thermogenesis	2/30	240/9308	1,80E+14	1,80E+14	4,56E+14	281715/614794	2
bta04927	Cortisol synthesis and secretion	1/30	67/9308	1,95E+14	1,95E+14	4,56E+14	281715	1
bta05031	Amphetamine addiction	1/30	69/9308	2,00E+14	2,00E+14	4,56E+14	281715	1
bta00562	Inositol phosphate metabolism	1/30	72/9308	2,08E+14	2,08E+14	4,56E+14	535487	1
bta04918	Thyroid hormone synthesis	1/30	76/9308	2,18E+14	2,18E+14	4,56E+14	281715	1
bta05100	Bacterial invasion of epithelial cells	1/30	76/9308	2,18E+14	2,18E+14	4,56E+14	280794	1
bta03018	RNA degradation	1/30	78/9308	2,23E+14	2,23E+14	4,56E+14	512320	1
bta04721	Synaptic vesicle cycle	1/30	79/9308	2,26E+14	2,26E+14	4,56E+14	530692	1
bta04917	Prolactin signaling pathway	1/30	84/9308	2,38E+14	2,38E+14	4,56E+14	281422	1
bta04911	Insulin secretion	1/30	85/9308	2,41E+14	2,41E+14	4,56E+14	281715	1
bta01232	Nucleotide metabolism	1/30	86/9308	2,43E+14	2,43E+14	4,56E+14	512320	1
bta04662	B cell receptor signaling pathway	1/30	87/9308	2,46E+14	2,46E+14	4,56E+14	783725	1
bta04211	Longevity regulating pathway	1/30	90/9308	2,53E+13	2,53E+13	4,56E+14	281715	1
bta04512	ECM-receptor interaction	1/30	90/9308	2,53E+13	2,53E+13	4,56E+14	280794	1
bta05410	Hypertrophic cardiomyopathy	1/30	92/9308	2,58E+14	2,58E+14	4,56E+14	497015	1
bta04260	Cardiac muscle contraction	1/30	93/9308	2,60E+14	2,60E+14	4,56E+14	497015	1

bta05222	Small cell lung cancer	1/30	94/9308	2,63E+14	2,63E+14	4,56E+14	280794	1
bta03008	Ribosome biogenesis in eukaryotes	1/30	95/9308	2,65E+14	2,65E+14	4,56E+14	100126828	1
bta04925	Aldosterone synthesis and secretion	1/30	96/9308	2,68E+14	2,68E+14	4,56E+14	281715	1
bta04070	Phosphatidylinositol signaling system	1/30	97/9308	2,70E+14	2,70E+14	4,56E+14	535487	1
bta05414	Dilated cardiomyopathy	1/30	99/9308	2,75E+13	2,75E+13	4,56E+14	497015	1
bta04916	Melanogenesis	1/30	102/9308	2,82E+14	2,82E+14	4,56E+14	281715	1
bta04922	Glucagon signaling pathway	1/30	103/9308	2,84E+14	2,84E+14	4,56E+14	281715	1
bta04933	AGE-RAGE signaling pathway in diabetic complications	1/30	103/9308	2,84E+14	2,84E+14	4,56E+14	280794	1
bta04928	Parathyroid hormone synthesis, secretion and action	1/30	105/9308	2,89E+14	2,89E+14	4,56E+14	281715	1
bta04931	Insulin resistance	1/30	110/9308	3,00E+14	3,00E+14	4,66E+14	281715	1
bta04725	Cholinergic synapse	1/30	114/9308	3,09E+13	3,09E+13	4,72E+14	281715	1
bta05146	Amoebiasis	1/30	117/9308	3,16E+14	3,16E+14	4,72E+14	280794	1
bta04935	Growth hormone synthesis, secretion and action	1/30	119/9308	3,21E+13	3,21E+13	4,72E+14	281715	1
bta04611	Platelet activation	1/30	122/9308	3,27E+14	3,27E+14	4,72E+14	783470	1
bta04152	AMPK signaling pathway	1/30	124/9308	3,32E+14	3,32E+14	4,72E+14	281715	1
bta04668	TNF signaling pathway	1/30	126/9308	3,36E+14	3,36E+14	4,72E+14	281715	1
bta04926	Relaxin signaling pathway	1/30	131/9308	3,47E+14	3,47E+14	4,79E+14	281715	1
bta04728	Dopaminergic synapse	1/30	134/9308	3,53E+13	3,53E+13	4,80E+14	281715	1
bta04915	Estrogen signaling pathway	1/30	138/9308	3,62E+14	3,62E+14	4,84E+14	281715	1
bta01240	Biosynthesis of cofactors	1/30	152/9308	3,90E+14	3,90E+14	5,15E+14	286864	1
bta05135	Yersinia infection	1/30	157/9308	4,00E+14	4,00E+14	5,15E+14	280794	1
bta04934	Cushing syndrome	1/30	158/9308	4,02E+14	4,02E+14	5,15E+14	281715	1
bta04218	Cellular senescence	1/30	165/9308	4,16E+14	4,16E+14	5,25E+14	518502	1
bta04022	cGMP-PKG signaling pathway	1/30	171/9308	4,27E+14	4,27E+14	5,32E+14	281715	1
bta05161	Hepatitis B	1/30	177/9308	4,38E+14	4,38E+14	5,38E+14	281715	1
bta05202	Transcriptional misregulation in cancer	1/30	200/9308	4,79E+14	4,79E+14	5,73E+14	511037	1
bta04814	Motor proteins	1/30	202/9308	4,83E+13	4,83E+13	5,73E+14	497015	1
bta05205	Proteoglycans in cancer	1/30	204/9308	4,86E+12	4,86E+12	5,73E+14	280794	1
bta04630	JAK-STAT signaling pathway	1/30	212/9308	5,00E+14	5,00E+14	5,76E+14	281422	1
bta05207	Chemical carcinogenesis - receptor activation	1/30	216/9308	5,06E+14	5,06E+14	5,76E+14	281715	1
bta04015	Rap1 signaling pathway	1/30	217/9308	5,08E+14	5,08E+14	5,76E+14	783470	1
bta04810	Regulation of actin cytoskeleton	1/30	228/9308	5,25E+14	5,25E+14	5,88E+14	280794	1
bta05034	Alcoholism	1/30	232/9308	5,32E+14	5,32E+14	5,88E+14	281715	1
bta05417	Lipid and atherosclerosis	1/30	239/9308	5,42E+14	5,42E+14	5,90E+14	510745	1
bta05203	Viral carcinogenesis	1/30	242/9308	5,47E+14	5,47E+14	5,90E+14	281715	1
bta04024	cAMP signaling pathway	1/30	247/9308	5,54E+14	5,54E+14	5,90E+14	281715	1
bta05163	Human cytomegalovirus infection	1/30	251/9308	5,60E+14	5,60E+14	5,90E+14	281715	1
bta05020	Prion disease	1/30	282/9308	6,03E+14	6,03E+14	6,28E+14	281715	1
bta05016	Huntington disease	1/30	319/9308	6,49E+14	6,49E+14	6,68E+14	281715	1
bta04060	Cytokine-cytokine receptor interaction	1/30	333/9308	6,65E+14	6,65E+14	6,76E+14	281422	1
bta05014	Amyotrophic lateral sclerosis	1/30	383/9308	7,17E+14	7,17E+14	7,20E+14	507345	1
bta04080	Neuroactive ligand-receptor interaction	1/30	394/9308	7,27E+14	7,27E+14	7,23E+13	281422	1
bta05022	Pathways of neurodegeneration - multiple diseases	1/30	490/9308	8,03E+14	8,03E+14	7,89E+14	507345	1

[1]ID: Kegg pathway identifier

Table S6 – KEGG functional enrichment related to the Subcutaneous Fat Thickness (SFT) trait.

ID¹	Description	GeneRatio	BgRatio	p-value	p.adjust	q-value	geneID	Count
bta00350	Tyrosine metabolism	5/19	39/9308	1,10E+06	1,10E+06	2,90E+07	534808/509744/510551/520508/505515	5
bta00071	Fatty acid degradation	5/19	44/9308	2,07E+06	2,07E+06	2,90E+07	534808/509744/510551/520508/505515	5
bta00620	Pyruvate metabolism	5/19	44/9308	2,07E+06	2,07E+06	2,90E+07	534808/509744/510551/520508/505515	5
bta00982	Drug metabolism - cytochrome P450	5/19	62/9308	1,20E+07	1,20E+07	1,19E+08	534808/509744/510551/520508/505515	5
bta00010	Glycolysis / Gluconeogenesis	5/19	64/9308	1,42E+07	1,42E+07	1,19E+08	534808/509744/510551/520508/505515	5
bta00980	Metabolism of xenobiotics by cytochrome P450	5/19	67/9308	1,79E+07	1,79E+07	1,25E+08	534808/509744/510551/520508/505515	5
bta00830	Retinol metabolism	5/19	72/9308	2,57E+07	2,57E+07	1,55E+08	534808/509744/510551/520508/505515	5
bta04936	Alcoholic liver disease	5/19	154/9308	1,12E+07	1,12E+07	5,90E+09	534808/509744/510551/520508/505515	5
bta04150	mTOR signaling pathway	2/19	158/9308	4,05E+14	4,05E+14	1,90E+13	281751/533861	2
bta03060	Protein export	1/19	23/9308	4,60E+14	4,60E+14	1,94E+14	415113	1
bta04810	Regulation of actin cytoskeleton	2/19	228/9308	7,78E+14	7,78E+14	2,83E+14	613421/525628	2
bta03050	Proteasome	1/19	47/9308	9,18E+14	9,18E+14	2,83E+14	511207	1
bta04975	Fat digestion and absorption	1/19	52/9308	1,01E+14	1,01E+14	2,83E+14	280868	1
bta04340	Hedgehog signaling pathway	1/19	56/9308	1,08E+14	1,08E+14	2,83E+14	286821	1
bta05012	Parkinson disease	2/19	282/9308	1,12E+14	1,12E+14	2,83E+14	511207/415113	2
bta05020	Prion disease	2/19	282/9308	1,12E+14	1,12E+14	2,83E+14	511207/415113	2
bta05206	MicroRNAs in cancer	2/19	293/9308	1,19E+14	1,19E+14	2,83E+14	790997/791012	2
bta05217	Basal cell carcinoma	1/19	63/9308	1,21E+14	1,21E+14	2,83E+14	286821	1
bta04927	Cortisol synthesis and secretion	1/19	67/9308	1,28E+14	1,28E+14	2,84E+14	281948	1
bta04918	Thyroid hormone synthesis	1/19	76/9308	1,44E+14	1,44E+14	2,85E+14	415113	1
bta05100	Bacterial invasion of epithelial cells	1/19	76/9308	1,44E+14	1,44E+14	2,85E+14	613421	1
bta01521	EGFR tyrosine kinase inhibitor resistance	1/19	80/9308	1,51E+14	1,51E+14	2,85E+14	281751	1
bta04662	B cell receptor signaling pathway	1/19	87/9308	1,64E+14	1,64E+14	2,85E+14	533156	1
bta04211	Longevity regulating pathway	1/19	90/9308	1,69E+14	1,69E+14	2,85E+14	281751	1
bta04612	Antigen processing and presentation	1/19	91/9308	1,70E+14	1,70E+14	2,85E+14	415113	1
bta04666	Fc gamma R-mediated phagocytosis	1/19	97/9308	1,81E+14	1,81E+14	2,85E+14	613421	1
bta05014	Amyotrophic lateral sclerosis	2/19	383/9308	1,83E+14	1,83E+14	2,85E+14	511207/415113	2
bta04066	HIF-1 signaling pathway	1/19	110/9308	2,02E+14	2,02E+14	2,99E+14	281751	1
bta01200	Carbon metabolism	1/19	112/9308	2,06E+14	2,06E+14	2,99E+14	505515	1
bta04726	Serotonergic synapse	1/19	121/9308	2,20E+14	2,20E+14	3,09E+14	541200	1
bta04910	Insulin signaling pathway	1/19	138/9308	2,47E+14	2,47E+14	3,25E+14	281751	1
bta05017	Spinocerebellar ataxia	1/19	146/9308	2,60E+14	2,60E+14	3,25E+14	511207	1

bta05022	Pathways of neurodegeneration - multiple diseases	2/19	490/9308	2,64E+14	2,64E+14	3,25E+14	511207/415113	2
bta05226	Gastric cancer	1/19	153/9308	2,70E+14	2,70E+14	3,25E+14	286821	1
bta05135	Yersinia infection	1/19	157/9308	2,76E+14	2,76E+14	3,25E+14	613421	1
bta04934	Cushing syndrome	1/19	158/9308	2,78E+14	2,78E+14	3,25E+14	281948	1
bta03010	Ribosome	1/19	168/9308	2,93E+13	2,93E+13	3,25E+14	515534	1
bta04141	Protein processing in endoplasmic reticulum	1/19	175/9308	3,03E+14	3,03E+14	3,25E+14	415113	1
bta04530	Tight junction	1/19	178/9308	3,07E+14	3,07E+14	3,25E+14	613421	1
bta04360	Axon guidance	1/19	179/9308	3,09E+13	3,09E+13	3,25E+14	286821	1
bta05205	Proteoglycans in cancer	1/19	204/9308	3,44E+14	3,44E+14	3,53E+14	286821	1
bta05417	Lipid and atherosclerosis	1/19	239/9308	3,90E+13	3,90E+13	3,91E+14	415113	1
bta04144	Endocytosis	1/19	250/9308	4,04E+14	4,04E+14	3,95E+14	613421	1
bta05132	Salmonella infection	1/19	261/9308	4,18E+14	4,18E+14	3,95E+14	613421	1
bta04020	Calcium signaling pathway	1/19	265/9308	4,23E+14	4,23E+14	3,95E+14	541200	1
bta05171	Coronavirus disease - COVID-19	1/19	282/9308	4,43E+14	4,43E+14	4,05E+14	515534	1
bta05016	Huntington disease	1/19	319/9308	4,85E+14	4,85E+14	4,34E+14	511207	1
bta04151	PI3K-Akt signaling pathway	1/19	379/9308	5,46E+14	5,46E+14	4,78E+14	281751	1
bta04080	Neuroactive ligand-receptor interaction	1/19	394/9308	5,61E+14	5,61E+14	4,78E+14	541200	1
bta05010	Alzheimer disease	1/19	402/9308	5,68E+14	5,68E+14	4,78E+14	511207	1

[1]ID: Kegg pathway identifier

Table S7 – KEGG functional enrichment related to the Shear Force at 7 Days ageing (SF) trait.

ID	Description	GeneRatio	BgRatio	p-value	p.adjust	q-value	geneID	Count
bta00130	Ubiquinone and other terpenoid-quinone biosynthesis	1/20	11/9308	2,34E+14	2,34E+14	3,90E+14	504633	1
bta00450	Selenocompound metabolism	1/20	18/9308	3,80E+14	3,80E+14	3,90E+14	282388	1
bta00532	Glycosaminoglycan biosynthesis - chondroitin sulfate / dermatan sulfate	1/20	21/9308	4,42E+14	4,42E+14	3,90E+14	528860	1
bta00531	Glycosaminoglycan degradation	1/20	22/9308	4,63E+14	4,63E+14	3,90E+14	281230	1
bta04141	Protein processing in endoplasmic reticulum	2/20	175/9308	5,35E+14	5,35E+14	3,90E+14	282646/531964	2
bta00563	Glycosylphosphatidylinositol (GPI)-anchor biosynthesis	1/20	27/9308	5,65E+13	5,65E+13	3,90E+14	768015	1
bta04966	Collecting duct acid secretion	1/20	27/9308	5,65E+13	5,65E+13	3,90E+14	540113	1
bta01040	Biosynthesis of unsaturated fatty acids	1/20	30/9308	6,26E+14	6,26E+14	3,90E+14	617419	1
bta03440	Homologous recombination	1/20	42/9308	8,66E+14	8,66E+14	3,90E+14	504796	1
bta03410	Base excision repair	1/20	44/9308	9,05E+14	9,05E+14	3,90E+14	517825	1
bta04930	Type II diabetes mellitus	1/20	46/9308	9,44E+14	9,44E+14	3,90E+14	507041	1
bta01212	Fatty acid metabolism	1/20	58/9308	1,18E+14	1,18E+14	3,90E+14	617419	1
bta00561	Glycerolipid metabolism	1/20	68/9308	1,37E+14	1,37E+14	3,90E+14	519739	1
bta05211	Renal cell carcinoma	1/20	71/9308	1,42E+14	1,42E+14	3,90E+14	282711	1
bta04918	Thyroid hormone synthesis	1/20	76/9308	1,51E+14	1,51E+14	3,90E+14	282646	1
bta04721	Synaptic vesicle cycle	1/20	79/9308	1,57E+14	1,57E+14	3,90E+14	540113	1
bta03320	PPAR signaling pathway	1/20	84/9308	1,66E+14	1,66E+14	3,90E+14	617419	1
bta04917	Prolactin signaling pathway	1/20	84/9308	1,66E+14	1,66E+14	3,90E+14	514773	1
bta04612	Antigen processing and presentation	1/20	91/9308	1,79E+14	1,79E+14	3,90E+14	614382	1
bta04657	IL-17 signaling pathway	1/20	94/9308	1,84E+14	1,84E+14	3,90E+14	282646	1
bta04925	Aldosterone synthesis and secretion	1/20	96/9308	1,87E+14	1,87E+14	3,90E+14	507041	1
bta04666	Fc gamma R-mediated phagocytosis	1/20	97/9308	1,89E+14	1,89E+14	3,90E+14	507041	1
bta05215	Prostate cancer	1/20	98/9308	1,91E+14	1,91E+14	3,90E+14	282646	1
bta04933	AGE-RAGE signaling pathway in diabetic complications	1/20	103/9308	2,00E+13	2,00E+13	3,90E+14	507041	1
bta00564	Glycerophospholipid metabolism	1/20	104/9308	2,01E+14	2,01E+14	3,90E+14	519739	1
bta05323	Rheumatoid arthritis	1/20	106/9308	2,05E+14	2,05E+14	3,90E+14	540113	1
bta04750	Inflammatory mediator regulation of TRP channels	1/20	110/9308	2,12E+14	2,12E+14	3,90E+14	507041	1
bta04931	Insulin resistance	1/20	110/9308	2,12E+14	2,12E+14	3,90E+14	507041	1
bta04071	Sphingolipid signaling pathway	1/20	122/9308	2,32E+14	2,32E+14	3,90E+14	507041	1
bta04152	AMPK signaling pathway	1/20	124/9308	2,35E+14	2,35E+14	3,90E+14	617419	1
bta04910	Insulin signaling pathway	1/20	138/9308	2,58E+14	2,58E+14	3,90E+14	100139988	1
bta04915	Estrogen signaling pathway	1/20	138/9308	2,58E+14	2,58E+14	3,90E+14	282646	1
bta04270	Vascular smooth muscle contraction	1/20	140/9308	2,62E+14	2,62E+14	3,90E+14	507041	1
bta04371	Apelin signaling pathway	1/20	141/9308	2,63E+14	2,63E+14	3,90E+14	507041	1
bta00190	Oxidative phosphorylation	1/20	143/9308	2,67E+14	2,67E+14	3,90E+14	540113	1
bta05418	Fluid shear stress and atherosclerosis	1/20	145/9308	2,70E+14	2,70E+14	3,90E+14	282646	1
bta01240	Biosynthesis of cofactors	1/20	152/9308	2,81E+14	2,81E+14	3,90E+14	504633	1
bta04936	Alcoholic liver disease	1/20	154/9308	2,84E+14	2,84E+14	3,90E+14	617419	1
bta04150	mTOR signaling pathway	1/20	158/9308	2,90E+13	2,90E+13	3,90E+14	540113	1
bta04218	Cellular senescence	1/20	165/9308	3,01E+14	3,01E+14	3,90E+14	100139053	1
bta03010	Ribosome	1/20	168/9308	3,06E+14	3,06E+14	3,90E+14	613561	1
bta04022	cGMP-PKG signaling pathway	1/20	171/9308	3,10E+14	3,10E+14	3,90E+14	507041	1

bta04145	Phagosome	1/20	172/9308	3,12E+14	3,12E+14	3,90E+14	540113	1
bta05225	Hepatocellular carcinoma	1/20	174/9308	3,15E+14	3,15E+14	3,90E+14	282388	1
bta04530	Tight junction	1/20	178/9308	3,21E+14	3,21E+14	3,90E+14	507041	1
bta05205	Proteoglycans in cancer	1/20	204/9308	3,58E+14	3,58E+14	4,19E+14	281230	1
bta05152	Tuberculosis	1/20	205/9308	3,60E+14	3,60E+14	4,19E+14	614382	1
bta04630	JAK-STAT signaling pathway	1/20	212/9308	3,70E+14	3,70E+14	4,19E+14	514773	1
bta05207	Chemical carcinogenesis - receptor activation	1/20	216/9308	3,75E+14	3,75E+14	4,19E+14	282646	1
bta05166	Human T-cell leukemia virus 1 infection	1/20	238/9308	4,05E+14	4,05E+14	4,36E+14	614382	1
bta05417	Lipid and atherosclerosis	1/20	239/9308	4,06E+14	4,06E+14	4,36E+14	282646	1
bta05132	Salmonella infection	1/20	261/9308	4,34E+14	4,34E+14	4,57E+13	282646	1
bta05206	MicroRNAs in cancer	1/20	293/9308	4,73E+14	4,73E+14	4,88E+14	507041	1
bta05165	Human papillomavirus infection	1/20	352/9308	5,38E+14	5,38E+14	5,45E+14	540113	1
bta04151	PI3K-Akt signaling pathway	1/20	379/9308	5,65E+14	5,65E+14	5,62E+14	282646	1
bta05168	Herpes simplex virus 1 infection	1/20	416/9308	6,00E+14	6,00E+14	5,86E+14	505341	1

[1]ID: Kegg pathway identifier

Table S8 – KEGG functional enrichment related to the Intramuscular Fat (IMF) trait.

ID	Description	GeneRatio	BgRatio	p-value	p.adjust	q-value	geneID	Count
bta02010	ABC transporters	2/7	68/9308	1,08E+14	1,08E+14	1,02E+14	528068/101909228	2
bta04744	Phototransduction	1/7	28/9308	2,09E+14	2,09E+14	7,76E+14	281700	1
bta04136	Autophagy - other	1/7	33/9308	2,46E+14	2,46E+14	7,76E+14	534155	1
bta04115	p53 signaling pathway	1/7	80/9308	5,87E+14	5,87E+14	1,05E+14	534655	1
bta04660	T cell receptor signaling pathway	1/7	109/9308	7,92E+14	7,92E+14	1,05E+14	504733	1
bta04670	Leukocyte transendothelial migration	1/7	115/9308	8,34E+14	8,34E+14	1,05E+14	504782	1
bta04380	Osteoclast differentiation	1/7	138/9308	9,93E+14	9,93E+14	1,05E+14	504733	1
bta04140	Autophagy - animal	1/7	145/9308	1,04E+14	1,04E+14	1,05E+14	534155	1
bta04120	Ubiquitin mediated proteolysis	1/7	150/9308	1,08E+14	1,08E+14	1,05E+14	534655	1
bta04022	cGMP-PKG signaling pathway	1/7	171/9308	1,22E+14	1,22E+14	1,05E+14	281700	1
bta04310	Wnt signaling pathway	1/7	172/9308	1,22E+14	1,22E+14	1,05E+14	534655	1
bta04024	cAMP signaling pathway	1/7	247/9308	1,72E+13	1,72E+13	1,36E+14	281700	1

[1]ID: Kegg pathway identifier

Supplementary Archive 3

Table S9 – GO Enrichment Analysis for Biological processes (BP).

Cluster	Trait	ID¹	Description	GeneRatio	BgRatio	p-value	p.adjust	geneID	Count
1	REA	GO:0009615	response to virus	3/20	103/4543	9,75E+14	9,75E+14	281715/280873/280872	3
2	REA	GO:0006163	purine nucleotide metabolic process	3/20	118/4543	1,41E+14	1,41E+14	506343/510324/512320	3
3	REA	GO:0016197	endosomal transport	2/20	43/4543	1,49E+14	1,49E+14	507345/539393	2
4	REA	GO:0009152	purine ribonucleotide biosynthetic process	2/20	45/4543	1,63E+14	1,63E+14	506343/510324	2
5	REA	GO:0072521	purine-containing compound metabolic process	3/20	128/4543	1,75E+14	1,75E+14	506343/510324/512320	3
6	REA	GO:0006364	rRNA processing	2/20	48/4543	1,84E+14	1,84E+14	512320/518501	2
7	REA	GO:0009260	ribonucleotide biosynthetic process	2/20	50/4543	1,99E+14	1,99E+14	506343/510324	2
8	REA	GO:0009117	nucleotide metabolic process	3/20	136/4543	2,06E+14	2,06E+14	506343/510324/512320	3
9	REA	GO:0016072	rRNA metabolic process	2/20	51/4543	2,06E+13	2,06E+13	512320/518501	2
10	REA	GO:0046390	ribose phosphate biosynthetic process	2/20	51/4543	2,06E+13	2,06E+13	506343/510324	2
11	REA	GO:0006753	nucleoside phosphate metabolic process	3/20	139/4543	2,18E+14	2,18E+14	506343/510324/512320	3
12	REA	GO:1901135	carbohydrate derivative metabolic process	4/20	256/4543	2,33E+14	2,33E+14	506343/510324/282276/512320	4
13	REA	GO:0006164	purine nucleotide biosynthetic process	2/20	55/4543	2,38E+14	2,38E+14	506343/510324	2
14	REA	GO:0071345	cellular response to cytokine stimulus	3/20	147/4543	2,53E+14	2,53E+14	506343/280872/281422	3
15	REA	GO:0072522	purine-containing compound biosynthetic process	2/20	58/4543	2,63E+14	2,63E+14	506343/510324	2
16	REA	GO:0009144	purine nucleoside triphosphate metabolic process	2/20	61/4543	2,89E+14	2,89E+14	510324/512320	2
17	REA	GO:0055086	nucleobase-containing small molecule metabolic process	3/20	160/4543	3,15E+14	3,15E+14	506343/510324/512320	3
18	REA	GO:0009165	nucleotide biosynthetic process	2/20	64/4543	3,15E+13	3,15E+13	506343/510324	2
19	REA	GO:0034097	response to cytokine	3/20	161/4543	3,20E+14	3,20E+14	506343/280872/281422	3
20	REA	GO:1901293	nucleoside phosphate biosynthetic process	2/20	67/4543	3,43E+14	3,43E+14	506343/510324	2
21	REA	GO:0009141	nucleoside triphosphate metabolic process	2/20	69/4543	3,62E+14	3,62E+14	510324/512320	2
22	REA	GO:0016032	viral process	2/20	73/4543	4,02E+14	4,02E+14	281715/280872	2
23	REA	GO:0034976	response to endoplasmic reticulum stress	2/20	73/4543	4,02E+14	4,02E+14	507345/281715	2
24	REA	GO:0042254	ribosome biogenesis	2/20	73/4543	4,02E+14	4,02E+14	512320/518501	2
25	REA	GO:0009168	purine ribonucleoside monophosphate biosynthetic process	1/20	10/4543	4,32E+14	4,32E+14	506343	1
26	REA	GO:0045599	negative regulation of fat cell differentiation	1/20	10/4543	4,32E+14	4,32E+14	352960	1
27	REA	GO:0045740	positive regulation of DNA replication	1/20	10/4543	4,32E+14	4,32E+14	510324	1

28	REA	GO:0046471	phosphatidylglycerol metabolic process	1/20	10/4543	4,32E+14	4,32E+14	510324	1
29	REA	GO:0000002	mitochondrial genome maintenance	1/20	11/4543	4,74E+14	4,74E+14	510324	1
30	REA	GO:0009127	purine nucleoside monophosphate biosynthetic process	1/20	11/4543	4,74E+14	4,74E+14	506343	1
31	REA	GO:0032623	interleukin-2 production	1/20	11/4543	4,74E+14	4,74E+14	510324	1
32	REA	GO:0032663	regulation of interleukin-2 production	1/20	11/4543	4,74E+14	4,74E+14	510324	1
33	REA	GO:0036297	interstrand cross-link repair	1/20	11/4543	4,74E+14	4,74E+14	507345	1
34	REA	GO:0071168	protein localization to chromatin	1/20	11/4543	4,74E+14	4,74E+14	507345	1
35	REA	GO:0019221	cytokine-mediated signaling pathway	2/20	81/4543	4,85E+14	4,85E+14	280872/281422	2
409	SFT	GO:0072595	maintenance of protein localization in organelle	2/19	12/4543	1,07E+13	1,07E+13	281948/415113	2
410	SFT	GO:0032507	maintenance of protein location in cell	2/19	13/4543	1,26E+14	1,26E+14	281948/415113	2
411	SFT	GO:0045185	maintenance of protein location	2/19	16/4543	1,92E+14	1,92E+14	281948/415113	2
412	SFT	GO:0051651	maintenance of location in cell	2/19	46/4543	1,54E+14	1,54E+14	281948/415113	2
413	SFT	GO:0009725	response to hormone	3/19	137/4543	1,82E+14	1,82E+14	282260/404185/281948	3
414	SFT	GO:0007167	enzyme-linked receptor protein signaling pathway	3/19	153/4543	2,44E+13	2,44E+13	282260/404185/415113	3
415	SFT	GO:0043434	response to peptide hormone	2/19	67/4543	3,12E+13	3,12E+13	282260/404185	2
416	SFT	GO:0009719	response to endogenous stimulus	4/19	296/4543	3,14E+14	3,14E+14	282260/404185/281948/415113	4
417	SFT	GO:0051235	maintenance of location	2/19	73/4543	3,65E+14	3,65E+14	281948/415113	2
418	SFT	GO:0048870	cell motility	4/19	311/4543	3,68E+14	3,68E+14	282651/404185/281537/415113	4
419	SFT	GO:1901652	response to peptide	2/19	77/4543	4,03E+14	4,03E+14	282260/404185	2
420	SFT	GO:0003016	respiratory system process	1/19	10/4543	4,11E+14	4,11E+14	505515	1
421	SFT	GO:0022602	ovulation cycle process	1/19	10/4543	4,11E+14	4,11E+14	281948	1
422	SFT	GO:0042104	positive regulation of activated T cell proliferation	1/19	10/4543	4,11E+14	4,11E+14	282260	1
423	SFT	GO:0045047	protein targeting to ER	1/19	10/4543	4,11E+14	4,11E+14	415113	1
424	SFT	GO:0045599	negative regulation of fat cell differentiation	1/19	10/4543	4,11E+14	4,11E+14	511899	1
425	SFT	GO:0046580	negative regulation of Ras protein signal transduction	1/19	10/4543	4,11E+14	4,11E+14	533861	1
426	SFT	GO:0051055	negative regulation of lipid biosynthetic process	1/19	10/4543	4,11E+14	4,11E+14	511899	1
427	SFT	GO:0070828	heterochromatin organization	1/19	10/4543	4,11E+14	4,11E+14	281537	1
428	SFT	GO:0072599	establishment of protein localization to endoplasmic reticulum	1/19	10/4543	4,11E+14	4,11E+14	415113	1
429	SFT	GO:0006984	ER-nucleus signaling pathway	1/19	11/4543	4,51E+14	4,51E+14	415113	1
430	SFT	GO:0014812	muscle cell migration	1/19	11/4543	4,51E+14	4,51E+14	404185	1
431	SFT	GO:0042472	inner ear morphogenesis	1/19	11/4543	4,51E+14	4,51E+14	511899	1

432	SFT	GO:0048662	negative regulation of smooth muscle cell proliferation	1/19	11/4543	4,51E+14	4,51E+14	404185	1
433	SFT	GO:0051058	negative regulation of small GTPase mediated signal transduction	1/19	11/4543	4,51E+14	4,51E+14	533861	1
434	SFT	GO:0060021	roof of mouth development	1/19	11/4543	4,51E+14	4,51E+14	511899	1
435	SFT	GO:0065002	intracellular protein transmembrane transport	1/19	11/4543	4,51E+14	4,51E+14	415113	1
436	SFT	GO:1904888	cranial skeletal system development	1/19	11/4543	4,51E+14	4,51E+14	511899	1
437	SFT	GO:0007568	aging	1/19	12/4543	4,91E+14	4,91E+14	281948	1
438	SFT	GO:0032527	protein exit from endoplasmic reticulum	1/19	12/4543	4,91E+14	4,91E+14	511899	1
439	SFT	GO:0042149	cellular response to glucose starvation	1/19	12/4543	4,91E+14	4,91E+14	415113	1
440	SFT	GO:0042698	ovulation cycle	1/19	12/4543	4,91E+14	4,91E+14	281948	1
441	SFT	GO:0046006	regulation of activated T cell proliferation	1/19	12/4543	4,91E+14	4,91E+14	282260	1
442	SFT	GO:0048661	positive regulation of smooth muscle cell proliferation	1/19	12/4543	4,91E+14	4,91E+14	404185	1
443	SFT	GO:0071825	protein-lipid complex subunit organization	1/19	12/4543	4,91E+14	4,91E+14	280868	1
444	SFT	GO:0071827	plasma lipoprotein particle organization	1/19	12/4543	4,91E+14	4,91E+14	280868	1
445	SFT	GO:0120009	intermembrane lipid transfer	1/19	12/4543	4,91E+14	4,91E+14	280868	1
446	SFT	GO:1900407	regulation of cellular response to oxidative stress	1/19	12/4543	4,91E+14	4,91E+14	533861	1
447	SFT	GO:1901998	toxin transport	1/19	12/4543	4,91E+14	4,91E+14	415113	1
934	SF	GO:0044265	cellular macromolecule catabolic process	3/12	203/4543	1,43E+14	1,43E+14	282646/281230/514773	3
935	SF	GO:0030163	protein catabolic process	3/12	213/4543	1,63E+14	1,63E+14	282646/281230/514773	3
936	SF	GO:0043161	proteasome-mediated ubiquitin-dependent protein catabolic process	2/12	88/4543	2,16E+14	2,16E+14	282646/514773	2
937	SF	GO:0010972	negative regulation of G2/M transition of mitotic cell cycle	1/12	10/4543	2,61E+14	2,61E+14	504796	1
938	SF	GO:1902750	negative regulation of cell cycle G2/M phase transition	1/12	10/4543	2,61E+14	2,61E+14	504796	1
939	SF	GO:0042181	ketone biosynthetic process	1/12	11/4543	2,87E+14	2,87E+14	504633	1
940	SF	GO:0044773	mitotic DNA damage checkpoint signaling	1/12	11/4543	2,87E+14	2,87E+14	504796	1
941	SF	GO:0044774	mitotic DNA integrity checkpoint signaling	1/12	11/4543	2,87E+14	2,87E+14	504796	1
942	SF	GO:0001704	formation of primary germ layer	1/12	12/4543	3,13E+14	3,13E+14	282388	1
943	SF	GO:0032527	protein exit from endoplasmic reticulum	1/12	12/4543	3,13E+14	3,13E+14	282646	1
944	SF	GO:0035304	regulation of protein dephosphorylation	1/12	12/4543	3,13E+14	3,13E+14	282646	1
945	SF	GO:0010498	proteasomal protein catabolic process	2/12	111/4543	3,33E+14	3,33E+14	282646/514773	2
946	SF	GO:0031122	cytoplasmic microtubule organization	1/12	13/4543	3,38E+14	3,38E+14	617723	1

947	SF	GO:0046683	response to organophosphorus	1/12	13/4543	3,38E+14	3,38E+14	282646	1
948	SF	GO:0050732	negative regulation of peptidyl-tyrosine phosphorylation	1/12	13/4543	3,38E+14	3,38E+14	514773	1
949	SF	GO:1901661	quinone metabolic process	1/12	13/4543	3,38E+14	3,38E+14	504633	1
950	SF	GO:0009057	macromolecule catabolic process	3/12	288/4543	3,62E+14	3,62E+14	282646/281230/514773	3
951	SF	GO:0006029	proteoglycan metabolic process	1/12	14/4543	3,64E+14	3,64E+14	281230	1
952	SF	GO:0008299	isoprenoid biosynthetic process	1/12	14/4543	3,64E+14	3,64E+14	504633	1
953	SF	GO:0010389	regulation of G2/M transition of mitotic cell cycle	1/12	14/4543	3,64E+14	3,64E+14	504796	1
954	SF	GO:0010921	regulation of phosphatase activity	1/12	14/4543	3,64E+14	3,64E+14	282646	1
955	SF	GO:0032436	positive regulation of proteasomal ubiquitin-dependent protein catabolic process	1/12	14/4543	3,64E+14	3,64E+14	514773	1
956	SF	GO:0061097	regulation of protein tyrosine kinase activity	1/12	15/4543	3,90E+14	3,90E+14	514773	1
957	SF	GO:0099175	regulation of postsynapse organization	1/12	15/4543	3,90E+14	3,90E+14	617723	1
958	SF	GO:2000060	positive regulation of ubiquitin-dependent protein catabolic process	1/12	15/4543	3,90E+14	3,90E+14	514773	1
959	SF	GO:0042058	regulation of epidermal growth factor receptor signaling pathway	1/12	16/4543	4,15E+14	4,15E+14	514773	1
960	SF	GO:1902749	regulation of cell cycle G2/M phase transition	1/12	16/4543	4,15E+14	4,15E+14	504796	1
961	SF	GO:0000077	DNA damage checkpoint signaling	1/12	17/4543	4,40E+14	4,40E+14	504796	1
962	SF	GO:0014074	response to purine-containing compound	1/12	17/4543	4,40E+14	4,40E+14	282646	1
963	SF	GO:0030433	ubiquitin-dependent ERAD pathway	1/12	17/4543	4,40E+14	4,40E+14	282646	1
964	SF	GO:1901184	regulation of ERBB signaling pathway	1/12	17/4543	4,40E+14	4,40E+14	514773	1
965	SF	GO:0031570	DNA integrity checkpoint signaling	1/12	18/4543	4,66E+14	4,66E+14	504796	1
966	SF	GO:1901565	organonitrogen compound catabolic process	3/12	320/4543	4,73E+14	4,73E+14	282646/281230/514773	3
967	SF	GO:0006511	ubiquitin-dependent protein catabolic process	2/12	135/4543	4,76E+14	4,76E+14	282646/514773	2
968	SF	GO:0019941	modification-dependent protein catabolic process	2/12	136/4543	4,82E+14	4,82E+14	282646/514773	2
969	SF	GO:0043632	modification-dependent macromolecule catabolic process	2/12	136/4543	4,82E+14	4,82E+14	282646/514773	2
970	SF	GO:0007173	epidermal growth factor receptor signaling pathway	1/12	19/4543	4,91E+14	4,91E+14	514773	1
971	SF	GO:0007498	mesoderm development	1/12	19/4543	4,91E+14	4,91E+14	282388	1
1187	IMF	GO:0010972	negative regulation of G2/M transition of mitotic cell cycle	1/5	10/4543	1,10E+14	1,10E+14	616784	1

1188	IMF	GO:1902750	negative regulation of cell cycle G2/M phase transition	1/5	10/4543	1,10E+14	1,10E+14	616784	1
1189	IMF	GO:0031113	regulation of microtubule polymerization	1/5	11/4543	1,21E+14	1,21E+14	534203	1
1190	IMF	GO:0065002	intracellular protein transmembrane transport	1/5	11/4543	1,21E+14	1,21E+14	541085	1
1191	IMF	GO:0031122	cytoplasmic microtubule organization	1/5	13/4543	1,42E+14	1,42E+14	534203	1
1192	IMF	GO:0010389	regulation of G2/M transition of mitotic cell cycle	1/5	14/4543	1,53E+14	1,53E+14	616784	1
1193	IMF	GO:0046785	microtubule polymerization	1/5	14/4543	1,53E+14	1,53E+14	534203	1
1194	IMF	GO:0032273	positive regulation of protein polymerization	1/5	15/4543	1,64E+12	1,64E+12	534203	1
1195	IMF	GO:2000736	regulation of stem cell differentiation	1/5	15/4543	1,64E+12	1,64E+12	533520	1
1196	IMF	GO:0071806	protein transmembrane transport	1/5	16/4543	1,75E+14	1,75E+14	541085	1
1197	IMF	GO:1902749	regulation of cell cycle G2/M phase transition	1/5	16/4543	1,75E+14	1,75E+14	616784	1
1198	IMF	GO:0050994	regulation of lipid catabolic process	1/5	17/4543	1,86E+14	1,86E+14	541085	1
1199	IMF	GO:0031110	regulation of microtubule polymerization or depolymerization	1/5	19/4543	2,07E+14	2,07E+14	534203	1
1200	IMF	GO:0006635	fatty acid beta-oxidation	1/5	20/4543	2,18E+14	2,18E+14	541085	1
1201	IMF	GO:0000086	G2/M transition of mitotic cell cycle	1/5	21/4543	2,29E+14	2,29E+14	616784	1
1202	IMF	GO:0009062	fatty acid catabolic process	1/5	22/4543	2,40E+14	2,40E+14	541085	1
1203	IMF	GO:0019217	regulation of fatty acid metabolic process	1/5	22/4543	2,40E+14	2,40E+14	541085	1
1204	IMF	GO:0031109	microtubule polymerization or depolymerization	1/5	23/4543	2,51E+14	2,51E+14	534203	1
1205	IMF	GO:0044839	cell cycle G2/M phase transition	1/5	23/4543	2,51E+14	2,51E+14	616784	1
1206	IMF	GO:0007093	mitotic cell cycle checkpoint signaling	1/5	24/4543	2,61E+14	2,61E+14	616784	1
1207	IMF	GO:0019395	fatty acid oxidation	1/5	26/4543	2,83E+14	2,83E+14	541085	1
1208	IMF	GO:0034440	lipid oxidation	1/5	27/4543	2,94E+14	2,94E+14	541085	1
1209	IMF	GO:0072329	monocarboxylic acid catabolic process	1/5	27/4543	2,94E+14	2,94E+14	541085	1
1210	IMF	GO:0010565	regulation of cellular ketone metabolic process	1/5	28/4543	3,05E+14	3,05E+14	541085	1
1211	IMF	GO:0000724	double-strand break repair via homologous recombination	1/5	29/4543	3,15E+14	3,15E+14	616784	1
1212	IMF	GO:0000725	recombinational repair	1/5	29/4543	3,15E+14	3,15E+14	616784	1
1213	IMF	GO:1901991	negative regulation of mitotic cell cycle phase transition	1/5	29/4543	3,15E+14	3,15E+14	616784	1
1214	IMF	GO:0000075	cell cycle checkpoint signaling	1/5	32/4543	3,47E+14	3,47E+14	616784	1
1215	IMF	GO:0010212	response to ionizing radiation	1/5	33/4543	3,58E+14	3,58E+14	616784	1
1216	IMF	GO:0070507	regulation of microtubule cytoskeleton organization	1/5	34/4543	3,69E+14	3,69E+14	534203	1

1217	IMF	GO:0030258	lipid modification	1/5	36/4543	3,90E+14	3,90E+14	541085	1
1218	IMF	GO:1902905	positive regulation of supramolecular fiber organization	1/5	36/4543	3,90E+14	3,90E+14	534203	1
1219	IMF	GO:0045930	negative regulation of mitotic cell cycle	1/5	37/4543	4,01E+14	4,01E+14	616784	1
1220	IMF	GO:0051495	positive regulation of cytoskeleton organization	1/5	40/4543	4,33E+14	4,33E+14	534203	1
1221	IMF	GO:0031334	positive regulation of protein-containing complex assembly	1/5	41/4543	4,43E+14	4,43E+14	534203	1
1222	IMF	GO:0032271	regulation of protein polymerization	1/5	41/4543	4,43E+14	4,43E+14	534203	1
1223	IMF	GO:1901988	negative regulation of cell cycle phase transition	1/5	42/4543	4,54E+14	4,54E+14	616784	1
1224	IMF	GO:0048863	stem cell differentiation	1/5	45/4543	4,86E+13	4,86E+13	533520	1

[1]ID: Gene Ontology (GO) identification

Table S10 – GO Enrichment Analysis for Cellular component (CC).

**Cluster**	**Trait**	**ID[1]**	**Description**	**GeneRatio**	**BgRatio**	**pvalue**	**p.adjust**	**geneID**	**Count**
1	REA	GO:0000315	organellar large ribosomal subunit	2/27	43/4712	2,47E+14	2,47E+14	614151/614906	2
2	REA	GO:0005762	mitochondrial large ribosomal subunit	2/27	43/4712	2,47E+14	2,47E+14	614151/614906	2
3	REA	GO:0015934	large ribosomal subunit	2/27	50/4712	3,27E+14	3,27E+14	614151/614906	2
71	SFT	GO:0008180	COP9 signalosome	1/18	10/4712	3,76E+14	3,76E+14	415113	1
72	SFT	GO:0019774	proteasome core complex, beta-subunit complex	1/18	10/4712	3,76E+14	3,76E+14	511207	1
73	SFT	GO:0008076	voltage-gated potassium channel complex	1/18	11/4712	4,13E+14	4,13E+14	281123	1
127	SF	GO:0033178	proton-transporting two-sector ATPase complex, catalytic domain	1/12	11/4712	2,77E+14	2,77E+14	540113	1
128	SF	GO:0099503	secretory vesicle	2/12	133/4712	4,34E+14	4,34E+14	540223/540113	2
129	SF	GO:0016529	sarcoplasmic reticulum	1/12	19/4712	4,74E+14	4,74E+14	282646	1
130	SF	GO:0043197	dendritic spine	1/12	19/4712	4,74E+14	4,74E+14	617723	1
131	SF	GO:0044309	neuron spine	1/12	19/4712	4,74E+14	4,74E+14	617723	1
132	SF	GO:0001650	fibrillar center	1/12	20/4712	4,98E+14	4,98E+14	282388	1
194	IMF	GO:0035861	site of double-strand break	1/6	29/4712	3,64E+14	3,64E+14	616784	1
195	IMF	GO:0090734	site of DNA damage	1/6	35/4712	4,38E+14	4,38E+14	616784	1
196	IMF	GO:0001750	photoreceptor outer segment	1/6	38/4712	4,74E+14	4,74E+14	281700	1
197	IMF	GO:0005777	peroxisome	1/6	39/4712	4,87E+13	4,87E+13	404131	1
198	IMF	GO:0042579	microbody	1/6	39/4712	4,87E+13	4,87E+13	404131	1

[1]ID: Gene Ontology (GO) identification

Table S11 – GO Enrichment Analysis for Molecular function (MF).

Cluster	Trait	ID[1]	Description	GeneRatio	BgRatio	p-value	p.adjust	geneID	Count
1	REA	GO:0008483	transaminase activity	2/21	13/3912	2,07E+14	2,07E+14	282276/521553	2
2	REA	GO:0016769	transferase activity, transferring nitrogenous groups	2/21	14/3912	2,40E+14	2,40E+14	282276/521553	2
3	REA	GO:0042803	protein homodimerization activity	5/21	193/3912	2,96E+14	2,96E+14	506343/497015/281715/ 282276/512320	5
4	REA	GO:0042802	identical protein binding	7/21	450/3912	7,00E+14	7,00E+14	506343/497015/281715/282276/ 512320/280872/517889	7
5	REA	GO:0046983	protein dimerization activity	5/21	277/3912	1,36E+14	1,36E+14	506343/497015/281715/282276/ 512320	5
6	REA	GO:0005085	guanyl-nucleotide exchange factor activity	2/21	34/3912	1,39E+14	1,39E+14	780881/539393	2
7	REA	GO:0140678	molecular function inhibitor activity	3/21	126/3912	2,84E+14	2,84E+14	780881/539393/352960	3
8	REA	GO:0003723	RNA binding	4/21	244/3912	3,81E+14	3,81E+14	540872/513629/512320/518501	4
9	REA	GO:0030695	GTPase regulator activity	2/21	66/3912	4,79E+14	4,79E+14	780881/539393	2
10	REA	GO:0060589	nucleoside-triphosphatase regulator activity	2/21	66/3912	4,79E+14	4,79E+14	780881/539393	2
91	SFT	GO:0008289	lipid binding	5/17	174/3912	6,57E+14	6,57E+14	280868/505515/511899/281948/ 533861	5
92	SFT	GO:0019838	growth factor binding	2/17	21/3912	3,56E+14	3,56E+14	282260/404185	2
93	SFT	GO:0015459	potassium channel regulator activity	1/17	10/3912	4,27E+14	4,27E+14	281123	1
94	SFT	GO:0120013	lipid transfer activity	1/17	10/3912	4,27E+14	4,27E+14	280868	1
95	SFT	GO:0008134	transcription factor binding	2/17	82/3912	4,82E+14	4,82E+14	281751/281948	2
186	SF	GO:0015035	protein-disulfide reductase activity	1/8	10/3912	2,03E+14	2,03E+14	282388	1
187	SF	GO:0097110	scaffold protein binding	1/8	10/3912	2,03E+14	2,03E+14	617723	1
188	SF	GO:0015036	disulfide oxidoreductase activity	1/8	11/3912	2,23E+14	2,23E+14	282388	1
189	SF	GO:0030165	PDZ domain binding	1/8	11/3912	2,23E+14	2,23E+14	617723	1
190	SF	GO:0031593	polyubiquitin modification-dependent protein binding	1/8	14/3912	2,83E+14	2,83E+14	504796	1
191	SF	GO:0071949	FAD binding	1/8	16/3912	3,23E+14	3,23E+14	282388	1
192	SF	GO:0016667	oxidoreductase activity, acting on a sulfur group of donors	1/8	18/3912	3,63E+14	3,63E+14	282388	1
193	SF	GO:0004601	peroxidase activity	1/8	19/3912	3,82E+14	3,82E+14	282388	1
194	SF	GO:0016684	oxidoreductase activity, acting on peroxide as acceptor	1/8	19/3912	3,82E+14	3,82E+14	282388	1

195	SF	GO:0004553	hydrolase activity, hydrolyzing O-glycosyl compounds	1/8	20/3912	4,02E+14	4,02E+14	281230	1
196	SF	GO:0016765	transferase activity, transferring alkyl or aryl (other than methyl) groups	1/8	22/3912	4,42E+14	4,42E+14	504633	1
197	SF	GO:0140662	ATP-dependent protein folding chaperone	1/8	22/3912	4,42E+14	4,42E+14	282646	1
198	SF	GO:0008194	UDP-glycosyltransferase activity	1/8	23/3912	4,61E+14	4,61E+14	523294	1
199	SF	GO:0019903	protein phosphatase binding	1/8	23/3912	4,61E+14	4,61E+14	282646	1
229	IMF	GO:0030551	cyclic nucleotide binding	1/3	14/3912	1,07E+14	1,07E+14	281700	1
230	IMF	GO:0099094	ligand-gated monoatomic cation channel activity	1/3	18/3912	1,37E+14	1,37E+14	281700	1
231	IMF	GO:0002020	protease binding	1/3	27/3912	2,06E+14	2,06E+14	541085	1
232	IMF	GO:0015276	ligand-gated monoatomic ion channel activity	1/3	30/3912	2,28E+14	2,28E+14	281700	1
233	IMF	GO:0022834	ligand-gated channel activity	1/3	30/3912	2,28E+14	2,28E+14	281700	1
234	IMF	GO:0004252	serine-type endopeptidase activity	1/3	40/3912	3,04E+13	3,04E+13	541085	1
235	IMF	GO:0008236	serine-type peptidase activity	1/3	44/3912	3,34E+14	3,34E+14	541085	1
236	IMF	GO:0017171	serine hydrolase activity	1/3	44/3912	3,34E+14	3,34E+14	541085	1
237	IMF	GO:0005261	monoatomic cation channel activity	1/3	49/3912	3,71E+14	3,71E+14	281700	1
238	IMF	GO:0022836	gated channel activity	1/3	56/3912	4,23E+14	4,23E+14	281700	1
239	IMF	GO:0022839	monoatomic ion gated channel activity	1/3	56/3912	4,23E+14	4,23E+14	281700	1

[1]ID: Gene Ontology (GO) identification
